# One-photon red light-triggered disassembly of small-molecule nanoparticles for drug delivery

**DOI:** 10.1186/s12951-021-01103-z

**Published:** 2021-11-04

**Authors:** Kaiqi Long, Han Han, Weirong Kang, Wen Lv, Lang Wang, Yufeng Wang, Liang Ge, Weiping Wang

**Affiliations:** 1 State Key Laboratory of Pharmaceutical Biotechnology, The University of Hong Kong, Pokfulam, Hong Kong, China; 2 Department of Pharmacology and Pharmacy, Li Ka Shing Faculty of Medicine, The University of Hong Kong, Pokfulam, Hong Kong, China; 3 Dr Li Dak-Sum Research Centre, The University of Hong Kong, Pokfulam, Hong Kong, China; 4 State Key Laboratory of Natural Medicines and Department of Pharmaceutics, China Pharmaceutical University, Nanjing, Jiangsu China; 5 Department of Chemistry, The University of Hong Kong, Pokfulam, Hong Kong, China

**Keywords:** Photoresponsive drug delivery, Self-assembly, One-photon upconversion-like photolysis, Cancer therapy, Three-legged molecules

## Abstract

**Background:**

Photoresponsive drug delivery can achieve spatiotemporal control of drug accumulation at desired sites. Long-wavelength light is preferable owing to its deep tissue penetration and low toxicity. One-photon upconversion-like photolysis via triplet–triplet energy transfer (TTET) between photosensitizer and photoresponsive group enables the use of long-wavelength light to activate short-wavelength light-responsive groups. However, such process requires oxygen-free environment to achieve efficient photolysis due to the oxygen quenching of triplet excited states.

**Results:**

Herein, we report a strategy that uses red light to trigger disassembly of small-molecule nanoparticles by one-photon upconversion-like photolysis for cancer therapy. A photocleavable trigonal molecule, BTAEA, self-assembled into nanoparticles and enclosed photosensitizer, PtTPBP. Such nanoparticles protected TTET-based photolysis from oxygen quenching in normoxia aqueous solutions, resulting in efficient red light-triggered BTAEA cleavage, dissociation of nanoparticles and subsequent cargo release. With paclitaxel as the model drug, the red light-triggered drug release system demonstrated promising anti-tumor efficacy both in vitro and in vivo.

**Conclusions:**

This study provides a practical reference for constructing photoresponsive nanocarriers based on the one-photon upconversion-like photolysis.

**Graphical Abstract:**

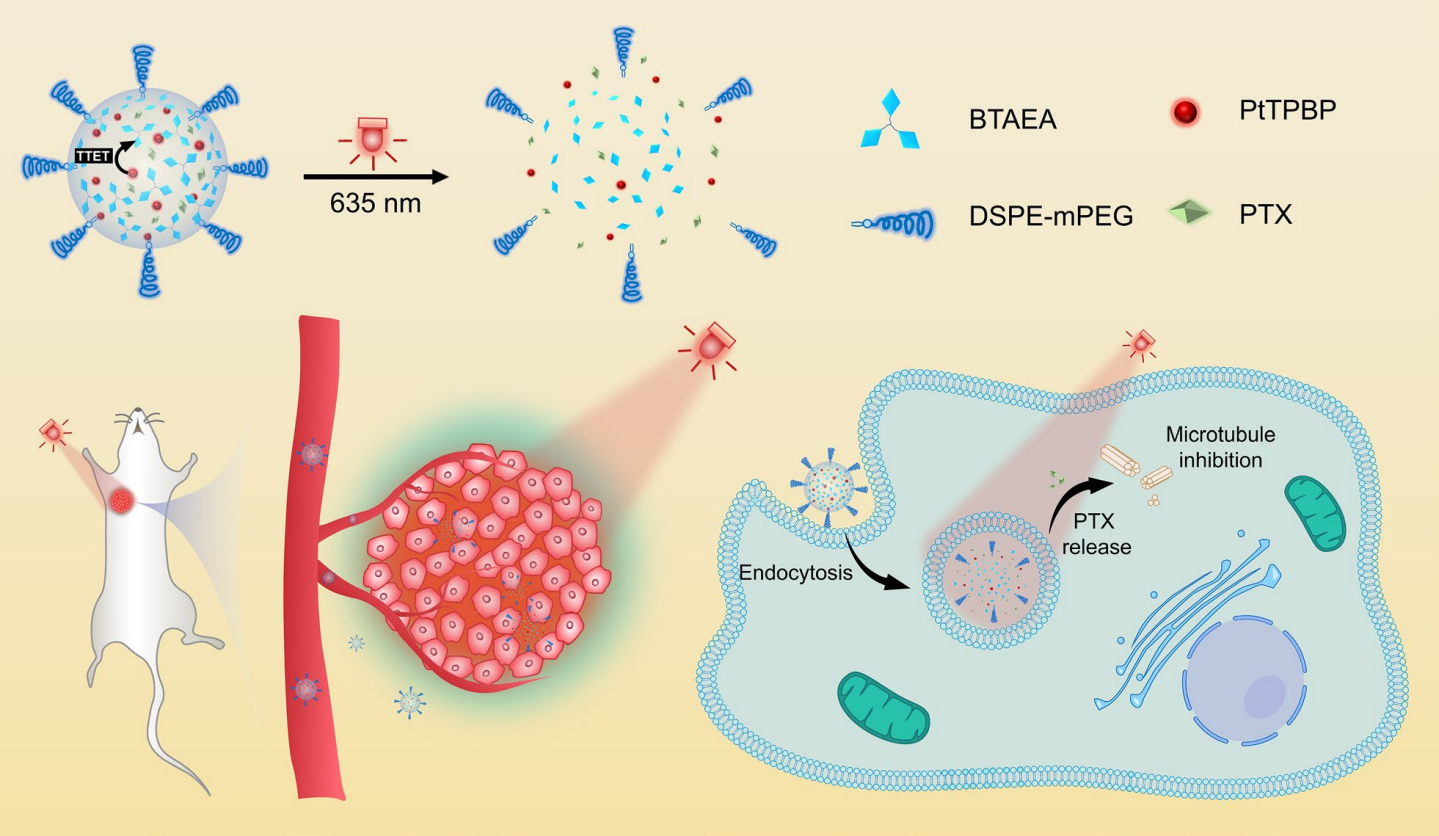

**Supplementary Information:**

The online version contains supplementary material available at 10.1186/s12951-021-01103-z.

## Introduction

Photoresponsive drug delivery systems open up new horizons for cancer treatments owing to their spatiotemporal control of drug accumulation in tumor tissues [1–4]. However, their in vivo applications are limited by the low tissue penetration and phototoxicity of light [5, 6]. Long-wavelength light, such as red or near-infrared light, is preferable because of its deep penetration [7–10]. Over the past few decades, substantial efforts have been focused on increasing the excitation wavelength of photoresponsive drug delivery systems. For example, photocleavable protecting groups (PPGs), such as boron-dipyrromethene (BODIPY) and cyanine, were designed to be photolyzed by long-wavelength light [11–14]. However, they usually have large π-conjugated structures that require sophisticated synthesis, and their photolysis quantum yields are usually low. Another strategy is to utilize multiphoton processes, such as two-photon excitation and upconversion luminescence (UCL) [15, 16]. Two-photon excitation of PPGs requires high-power femtosecond pump lasers that may generate heat and damage tissues [17]. UCL systems, including triplet–triplet annihilation systems and lanthanide-doped upconversion nanoparticles, rely on energy transfer processes [18, 19]. The luminescence quantum yields of UCL systems are theoretically restricted to be less than 0.5, and energy dissipation during multiple steps of energy transfer leads to low photolysis efficiency [17]. To date, developing strategies with high photolysis efficiency upon long-wavelength light irradiation remains a major challenge.

In our previous study, a one-photon upconversion-like process realized efficient photolysis of a green light-responsive prodrug, BODIPY-chlorambucil (BC), upon red-light irradiation [8, 20]. A photosensitizer, platinum (II) tetraphenyltetrabenzoporphyrin (PtTPBP), could harvest energy upon 635 nm red-light irradiation and subsequently activate BC prodrug through TTET process. Such process requires oxygen-free environment to maximize the photolysis efficiency because oxygen can hinder TTET by quenching the excited triplet states of prodrug or photosensitizer. A further study on a polymeric PLA-PEG nanocarrier encapsulating prodrug and photosensitizer enabled red light-triggered prodrug photolysis in normoxia conditions [21]. However, the type of drugs that can be used in the system is limited, since the synthesis of light-responsive prodrugs requires the drugs to contain certain functional groups (e.g., carboxyl group).

**Fig. 1:**
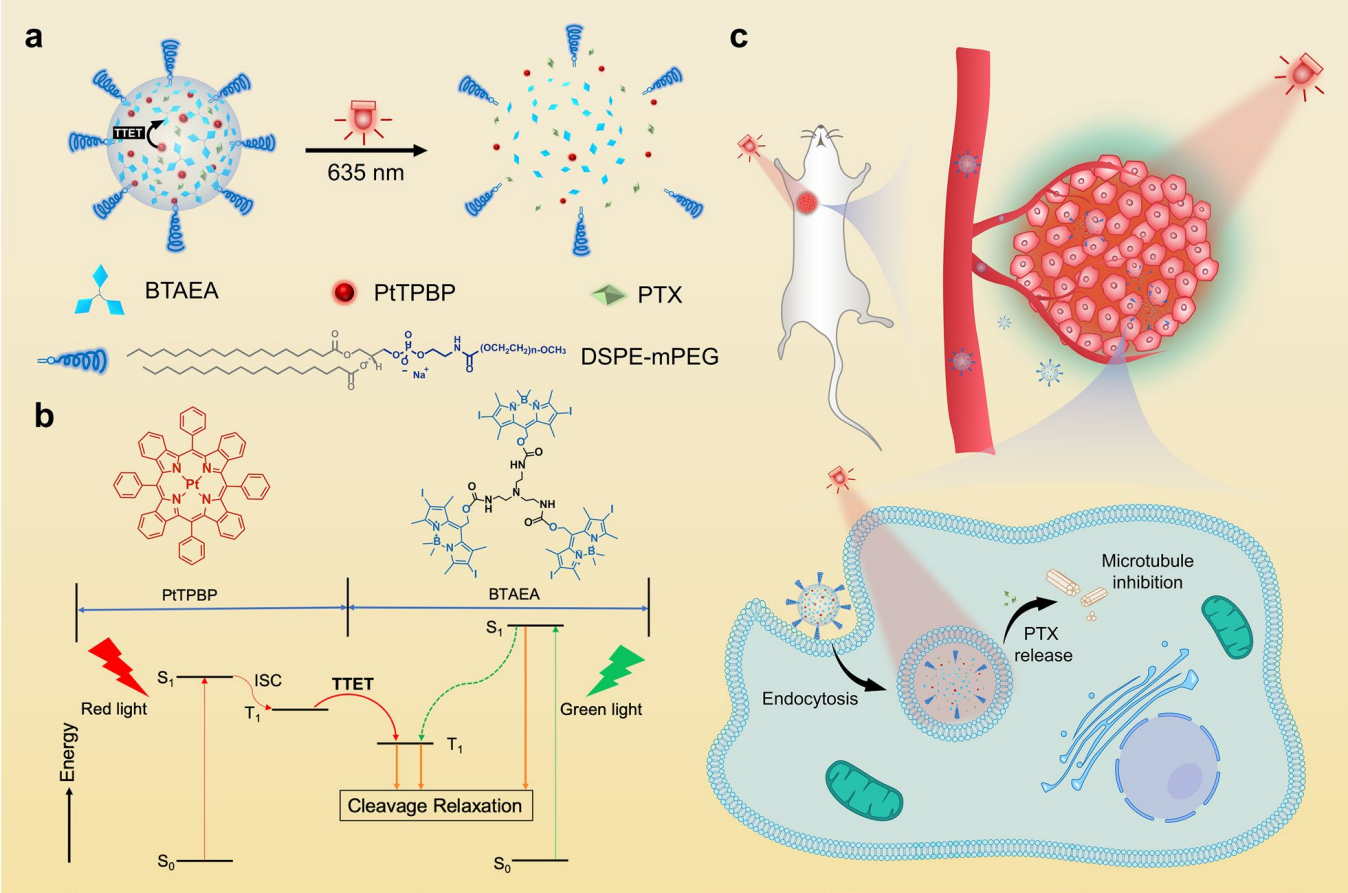
Schematic illustration of photoresponsive PTX/Pt/BTNP and its therapeutic effect. **a** Scheme of a red light-responsive nanoparticle consisting of BTAEA, PtTPBP, PTX and DSPE-mPEG. **b** Jablonski diagram demonstrating the mechanism of BTAEA photolysis via a one-photon TTET process (red lines) and the photolysis by direct excitation at the absorption wavelengths of BTAEA (green lines). **c** Scheme of red light-triggered drug release in vivo

In this study, we developed a drug delivery strategy that uses long-wavelength light to trigger disassembly of nanocarriers by the reported one-photon process. Compared with fabricating prodrug by conjugating drugs to PPGs, physically loading drugs in nanocarriers that can dissociate upon light and release drugs is more efficient and versatile [22, 23]. A trigonal molecule, (BODIPY)_3_-tris(2-aminoethyl)amine (BTAEA), was synthesized and demonstrated to be green-light cleavable. Our previous works confirmed that the distinctive trigonal structure allows BTAEA to self-assemble into nanoparticles via hydrophobic interactions and π–π interactions [24–27]. Photosensitizer (PtTPBP) and other cargos could be efficiently enclosed in nanoparticles by co-assembly. Upon red-light irradiation at 635 nm, the energy was harvested by PtTPBP and transferred to BTAEA via TTET, resulting in BTAEA photolysis (Fig. 1a, b). Notably, BTAEA molecule served as the energy acceptor as well as the backbone of the nanoparticles that protected the energy transfer from oxygen quenching. The release of cargos was verified after BTAEA photolysis and dissociation of the nanoparticles, resulting in efficient anti-tumor therapy both in vitro and in vivo (Fig. 1c). The simple design of the nanocarrier on the basis of small-molecule assembly and disassembly could be an inspiration for developing novel photoresponsive drug delivery systems.

## Results and discussions

### Preparation and characterization of nanoparticles

**Fig. 2:**
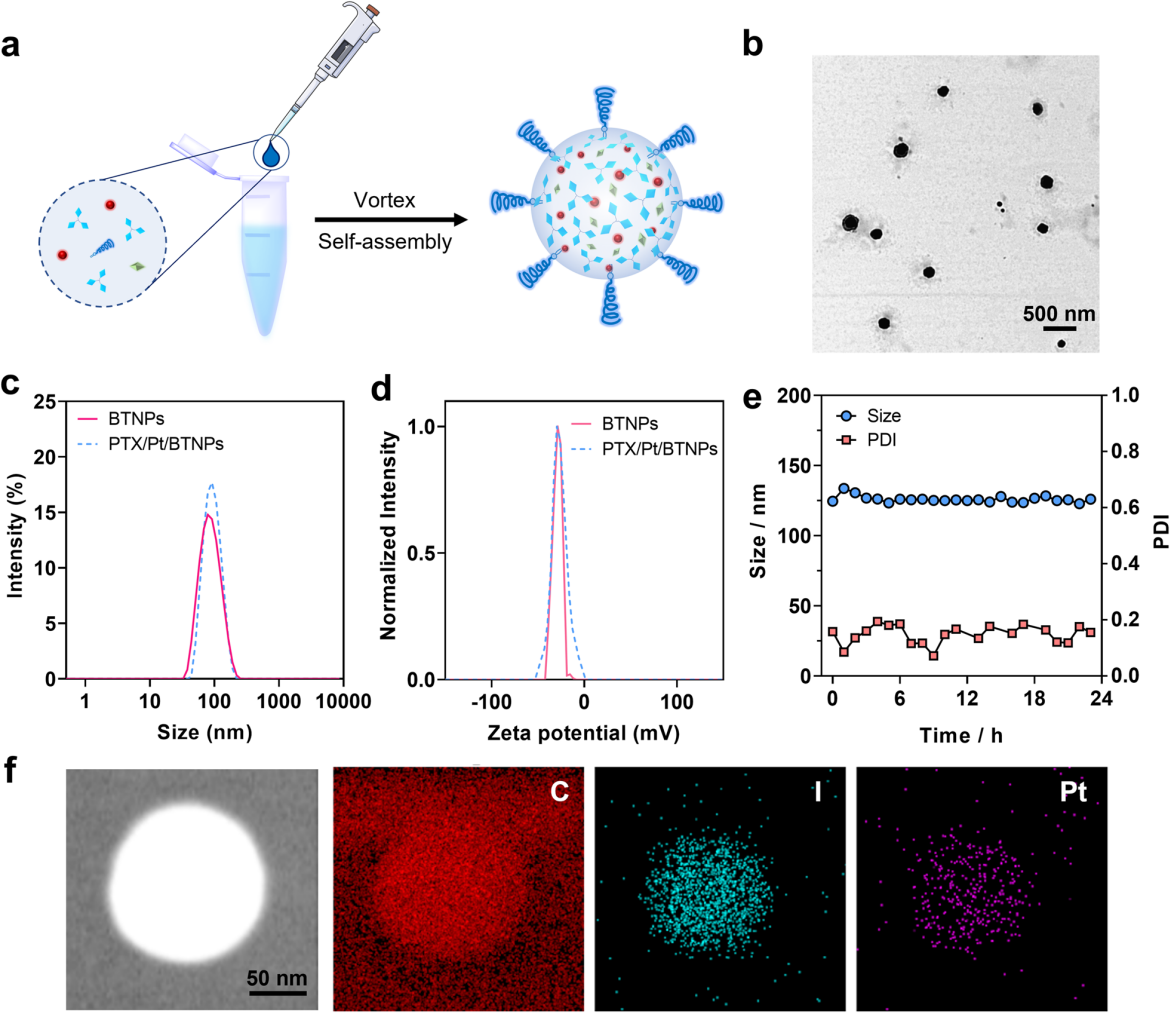
Fabrication and characterization of PTX/Pt/BTNPs. **a** Illustration of the preparation of PTX/Pt/BTNPs through flash nanoprecipitation method. **b** Representative TEM image of PTX/Pt/BTNPs. **c** DLS analysis of the size of BTNPs and PTX/Pt/BTNPs. **d** Zeta-potentials of BTNPs and PTX/Pt/BTNPs. **e** Stability of PTX/Pt/BTNPs in PBS for 24 h at 37 °C. **f** Elemental mapping images of the bright field, carbon (C), iodine (I) and platinum (Pt) of an individual PTX/Pt/BTNP

The photocleavable trigonal molecule, BTAEA, was synthesized (Additional file 1: Figure S1) and characterized by NMR and ESI–MS (Additional file 1: Figure S2–S7). Paclitaxel (PTX), a widely used anti-cancer drug, was chosen as a model drug for the formulation development [28, 29]. The self-assembled BTAEA nanoparticles loaded with PtTPBP and PTX were prepared by flash nanoprecipitation method (detailed procedures can be referred to Additional file 1: Experimental Section) (Fig. 2a) [25, 30]. The unentrapped drugs and photosensitizers were separated from the nanoparticles by centrifugation. The addition of 1,2-distearoyl-sn-glycero-3-phosphoethanolamine-*N*-[methoxy(polyethyleneglycol)-2000] (DSPE-mPEG_2000_) could improve the biocompatibility and extend the blood circulation time, which allows the system to passively accumulate in tumors [31–34]. The resulting nanoparticles, PTX/Pt/BTNPs, were collected and subjected to subsequent characterization.

The morphology and the size distribution of PTX/Pt/BTNPs were characterized by transmission electron microscopy (TEM) and dynamic light scattering (DLS), respectively. TEM images showed well-dispersed spherical nanoparticles (Fig. 2b). DLS data showed that PTX/Pt/BTNPs had an average diameter at about 95.7 nm with a polydispersity index (PDI) at 0.08 (Fig. 2c). The average zeta potential of the nanoparticles was −27.0 mV (Fig. 2d). Moreover, the size and zeta potential of the nanoparticles remained almost unchanged after loading with PTX and PtTPBP. PTX/Pt/BTNPs demonstrated desirable stability at 37 °C for at least 24 h, with negligible change of size and PDI in phosphate buffer saline (PBS) (Fig. 2e). Besides, the size of nanoparticles increased to about 200 nm after exposure to fetal bovine serum (FBS), and finally stabilized at about 160 nm within 24 h (Additional file 1: Figure S8a, b), which may be attributed to the formation of the dynamic protein corona on the surface of the nanoparticles. The aggregation of nanoparticles can be monitored by their absorbance, because the aggregated particles have higher light scattering than the non-aggregated particles [35, 36]. As showed in Additional file 1: Figure S8c, the absorbance values of PTX/Pt/BTNPs showed negligible change within 24 h in serum, indicated the good stability of PTX/Pt/BTNPs.

The composition of PTX/Pt/BTNP was investigated through TEM elemental mapping. As showed in Fig. 2f, both the iodine (I) from BTAEA and the platinum (Pt) from PtTPBP were homogenously distributed, implying the successfully encapsulation of PtTPBP in the nanoparticles. The proper ratio of BTAEA, PtTPBP, PTX and DSPE-mPEG were optimized in the nanoparticles. The drug loading of PTX was analyzed via high performance liquid chromatography (HPLC) based on the standard curves of BTAEA and PTX (Additional file 1: Figure S9). The amount of DSPE-mPEG was set as 20% (w/w) after comparing the size and PDI values of the nanoparticles with different ratios (5%, 10% and 20%) of DSPE-mPEG (Additional file 1: Figure S10a and b). The drug loading capacity of the nanoparticles increased from 5.6% to 13.1% while raising the weight ratio of PTX/BTAEA from 5 to 80% during the self-assembly process (Additional file 1: Figure S10c). The drug loading capacity slightly decreased to 12.6% while the weight ratio of PTX/BTAEA increased to 100%. Thus, the weight ratio of PTX/BTAEA was set as 80% in the following experiments. Besides, the ratio of PtTPBP was set as 10% (w/w) based on our previous study [20]. Thus, the formulation with optimized ratio of components (detailed in Additional file 1: Table S1) was used for the following evaluations.

### Photophysical characterization and photolysis study

**Fig. 3:**
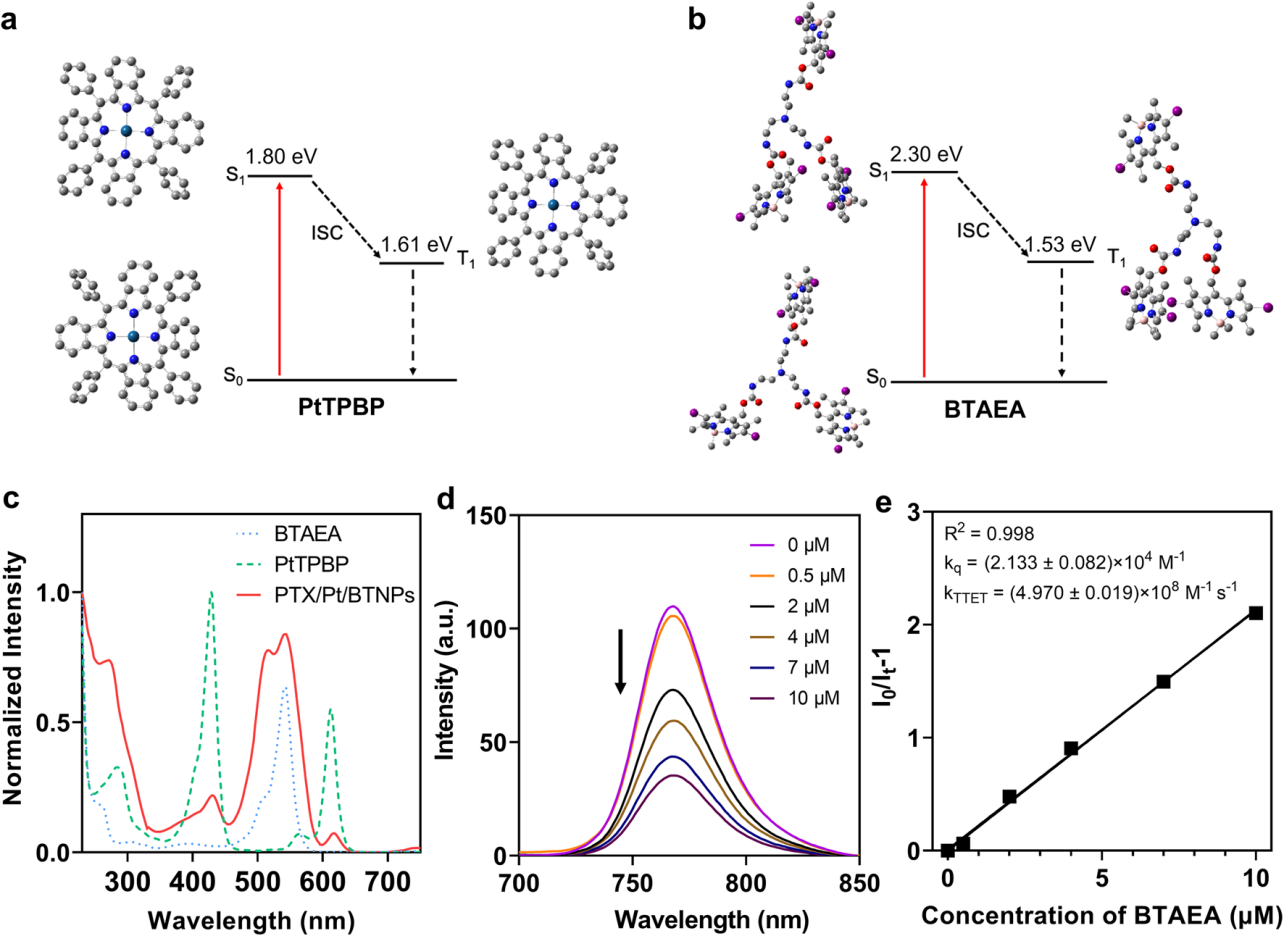
Photophysical characterization of PtTPBP, BTAEA and nanoparticles. Optimized excited state geometries and energy levels of BTAEA (**a**) and PtTPBP (**b**) determined at the B3LYP/6-31G(d) (LANL2DZ on I) level with Gaussian 16 software. **c** Normalized UV–vis absorption spectra of BTAEA, PtTPBP and PTX/Pt/BTNPs. **d** Phosphorescence intensity quenching of PtTPBP (10 μM) by BTAEA at different concentrations (0–10 μM) in N_2_-saturated methanol solution containing 10% toluene. **e** Stern−Volmer plot from phosphorescence quenching test and their linear fitness. (I_0_: phosphorescence intensity of PtTPBP in solution; I_t_: phosphorescence intensity of PtTPBP in the presence of BTAEA; *k*_*q*_: bimolecular quenching constant; *k*_*TTET*_: TTET rate constant)

Furthermore, the possibility of TTET between BTAEA and PtTPBP was demonstrated by calculation of their energy levels based on time-dependent density functional theory (Fig. 3a, b). As the results showed, the first excited singlet (S_1_) state of PtTPBP (1.80 eV) was lower than the S_1_ of BTAEA (2.30 eV), while the first excited triplet (T_1_) state of PtTPBP (1.60 eV) is slightly higher than the T_1_ state of BTAEA (1.53 eV). Thus, the energy transfer can occur between the T_1_ state of PtTPBP and the T_1_ state of BTAEA [20]. Besides, the lower S_1_ energy level of PtTPBP could enable the upconversion-like photolysis reaction of BTAEA (i.e., red light can cleave green light-responsive BTAEA). The absorption spectrum of PTX/Pt/BTNPs showed peaks at 542 nm and 618 nm, which are the characteristic peaks of BTAEA and PtTPBP, respectively (Fig. 3c). More importantly, the spectrum of PtTPBP revealed its red light-harvesting capacity at the range from 605 to 640 nm. To further verify the TTET process between PtTPBP and BTAEA, Stern–Volmer plots were determined by monitoring the phosphorescence of PtTPBP quenched by adding BTAEA in a N_2_-saturated solution (Fig. 3d, e) [37]. With the increase of BTAEA concentration, the phosphorescence of PtTPBP decreased, implying the energy transfer between these two molecules. The TTET rate constant (*k*_TTET_) was calculated to be (4.970 ± 0.019) × 10^8^ M^−1^ s^−1^, which is comparable to the reported values (about 10^9^ M^−1^ s^−1^) of TTET processes [20, 38, 39].

**Fig. 4:**
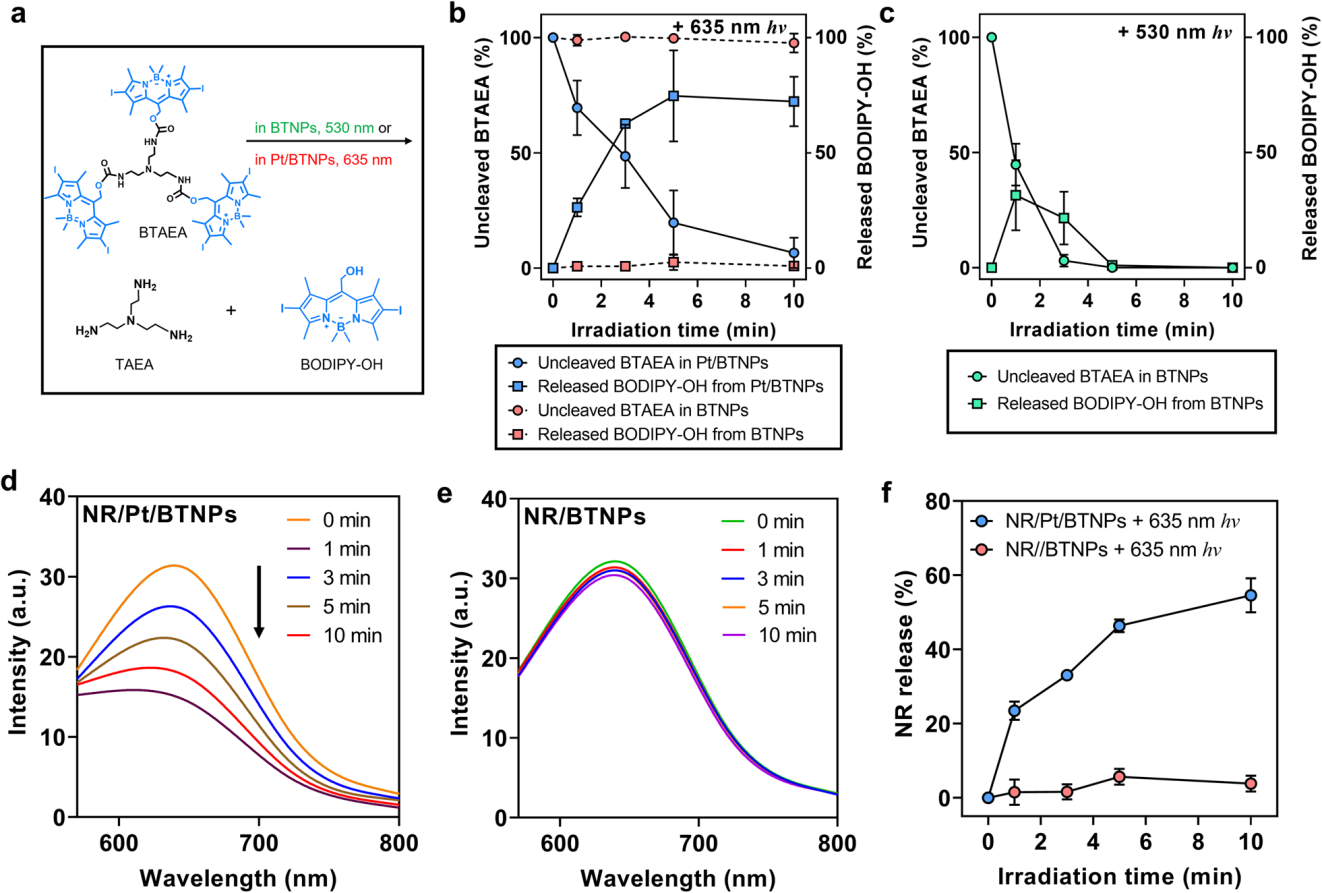
Photolysis study and light-triggered cargo release. **a** Photolysis reaction of BTAEA via direct excitation or one-photon TTET process. **b** Photolysis profile of BTAEA in BTNPs and Pt/BTNPs and the release of BODIPY-OH upon irradiation with red-light irradiation (635 nm) for different time periods (0–10 min) in normoxia aqueous solutions (n = 3). **c** Photolysis profile of BTAEA in BTNPs and the release of BODIPY-OH upon irradiation with green-light irradiation (530 nm) for different time periods (0–10 min) in normoxia aqueous solutions (n = 3). Fluorescence intensity of NR/Pt/BTNPs (**d**) and BTNPs (**e**) upon 635 nm light irradiation for different time periods (0–10 min). Excitation wavelength: 562 nm. **f** Calculated release percentage of Nile red from NR/Pt/BTNPs and NR/BTNPs under 635 nm red-light irradiation for different irradiation time periods (0–10 min). All light irradiations were conducted at 50 mW/cm^2^

The nanoparticles are proposed to protect BTAEA and PtTPBP from oxygen quenching and enable TTET between two molecules in normoxia conditions. To verify this hypothesis, the photolysis of BTAEA in the nanoparticles with or without PtTPBP (the nanoparticles abbreviated as Pt/BTNPs and BTNPs, respectively) in normoxia aqueous solutions was investigated under 530 nm (for direct excitation) or 635 nm (for one-photon TTET-based excitation) light irradiation. The photolysis reaction of BTAEA via direct excitation or one-photon TTET-based excitation was shown in Fig. 4a. It was observed that BTAEA can be photolyzed by 635 nm light irradiation in Pt/BTNPs. The concentrations of BTAEA were recorded by HPLC that can simultaneously detect BTAEA (R_t_ = 8.9 min) and its photolysis product BODIPY-OH (R_t_ = 6.8 min) (Fig. 4b; Additional file 1: Figure S11). The photolysis product was also confirmed by LC–MS (Additional file 1: Figure S12). As the irradiation time increased, the amount of BTAEA in Pt/BTNPs decreased continuously while its photolysis product BODIPY-OH increased up to ~75%. In contrast, BTAEA showed negligible photolysis in BTNPs that did not contain PtTPBP (Fig. 4b; Additional file 1: Figure S13), indicating that the red-light irradiation at 635 nm cannot directly cleave the BTAEA molecule. As a comparison, green-light irradiation at 530 nm rapidly triggered the photolysis of BTAEA in the absence of PtTPBP (Fig. 4c; Additional file 1: Figure S14), due to the direct excitation of BTAEA by 530 nm light irradiation. These results revealed that PtTPBP within the nanoparticles harvested low-energy photons and activated BTAEA via one-photon TTET process, resulting in long-wavelength light-triggered photocleavage of BTAEA.

The structural transformation of the PTX/Pt/BTNPs upon light irradiation and its subsequent payload release were investigated. As BTAEA is the dominant component in the nanoparticles, its photolysis may result in dissociation of the nanoparticles. Therefore, the size distribution and morphology were characterized after light irradiation. The TEM images showed the dissociation of nanoparticles upon light irradiation (Additional file 1: Figure S15a). From the DLS analysis, the PDI value of PTX/Pt/BTNPs remarkably increased from 0.1 to 0.6 after light irradiation, and the change of size distributions also indicated the formation of small fragments and aggregates during the period (Additional file 1: Figure S15b).

### Photo-triggered drug release performance

To visualize the payload release and cellular uptake behaviours of Pt/BTNPs, Nile red (NR), a fluorescent dye, was loaded in the nanoparticles (NR/Pt/BTNPs) as a model drug. NR is a reporter molecule that reveals the formation and disruption of nanoassemblies as it only has obvious fluorescence in the hydrophobic environment and the fluorescence decreases when NR is released in aqueous solutions [40, 41]. As demonstrated in Fig. 4d, the fluorescence of NR/Pt/BTNPs dramatically decreased upon red-light irradiation (635 nm) within 10 min, implying the release of enclosed NR. On the contrary, the nanoparticles without PtTPBP showed only minimal release of NR from BTNPs upon the red-light irradiation (Fig. 4e). The results confirmed that the red light could cleave BTAEA molecules in the nanoparticles in the presence of PtTPBP, leading to nanoparticle dissociation and triggering cargo release (Fig. 4f).

**Fig. 5:**
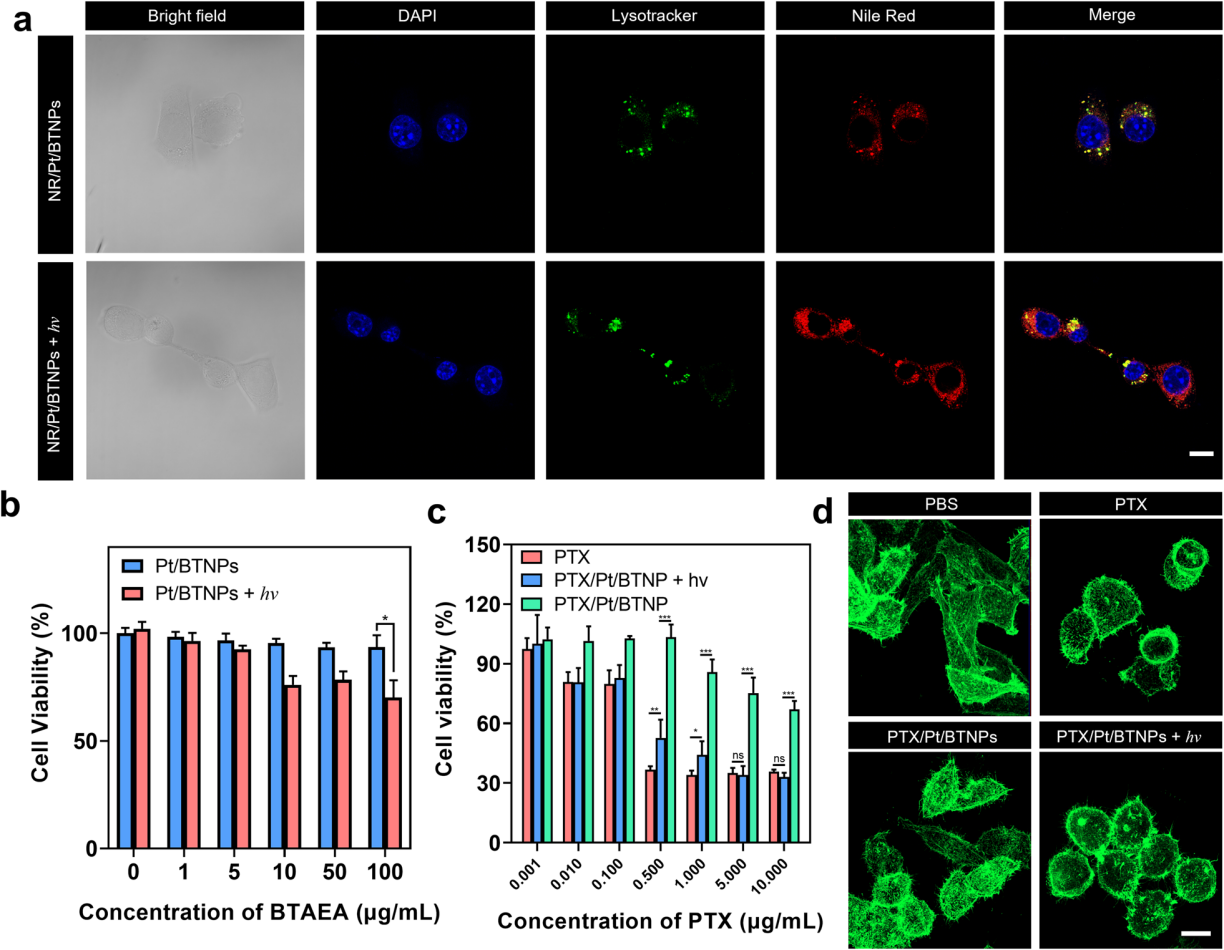
In vitro study of the light-triggered drug release performance and cytotoxicity. **a** CLSM images of 4T1 cells incubated with NR/Pt/BTNPs with or without light irradiation. Scale bar: 20 µm. **b** Cell viability of HUVECs treated with Pt/BTNPs with or without light irradiation (n = 5). **c** Cell viability of 4T1 cells treated with PTX/Pt/BTNPs with or without light irradiation (n = 5). **d** CLSM images showing the actin change of 4T1 cells. Cells were treated for 4 h and incubated in fresh cell culture media for another 24 h. Scale bar: 20 µm. All irradiations were done with a 635 nm laser (50 mW/cm^2^, 10 min)

The light-triggered NR release and the cellular uptake thereafter was investigated with murine breast cancer 4T1 cells by confocal laser scanning microscopy (CLSM). Lysosomes were labelled with LysoTracker^®^ Green DND-26. Figure 5a showed that NR/Pt/BTNPs were mostly co-localized with lysosomes after 4 h incubation without light irradiation since the nanoparticles were mostly taken up by endocytosis. In contrast, the co-localization significantly reduced while applying the red-light irradiation. The NR signal distributed in the cytoplasm, which can be explained by the light-triggered NR release from the nanoparticles and the efficient cellular uptake of free NR. The intracellular drug release evaluation indicated that the cargos in the nanoparticles, such as hydrophobic dye and drug, can be released upon light irradiation and diffused into cytoplasm as the result.

### Biocompatibility and in vitro therapeutic effect

The biocompatibility of drug-free nanoparticles, Pt/BTNPs, was investigated by determining the viability of human umbilical vein endothelial cells (HUVECs) after the treatment with Pt/BTNPs with or without light irradiation. Pt/BTNPs did not show notable toxicity at BTAEA concentrations up to 100 μg/mL (Fig. 5b) after 24 h incubation with HUVECs in dark, demonstrating desirable biocompatibility of the nanocarriers. Upon light irradiation at 635 nm, there was no obvious toxicity at up to 5 μg/mL of BTAEA, while the cytotoxicity slightly increased with the BTAEA concentration in the range of 10 to 100 μg/mL, which is probably due to the singlet oxygen (^1^O_2_) generated from Pt/BTNPs upon light irradiation. Given the light-responsive payload release of Pt/BTNPs, PTX was loaded as a model drug to explore the anti-tumor therapeutic effect*.* The light-triggered cytotoxicity of PTX/Pt/BTNPs was investigated on 4T1 cells. The viability of cells treated with PTX/Pt/BTNPs significantly decreased after the red-light irradiation at 635 nm (50 mW/cm^2^, 10 min) (IC_50_: 264.5 ng/mL) (Fig. 5c), which is similar to the free PTX-treated cells (IC_50_: 179.8 ng/mL). Nonetheless, the treatment without light irradiation showed comparable cell viability to the control group. Therefore, the light irradiation can successfully trigger sufficient PTX release and lead to cancer cell death. Moreover, the therapeutic effect of PTX on microtubules was evaluated by staining with Alexa Fluor 488-phalloidin. The cells treated with PTX/Pt/BTNPs plus light irradiation had small and impaired actin bundles and displayed a round shape, which is similar to the cells treated with free PTX at the same concentration. In contrast, the cells treated with PBS or the nanoparticles without light irradiation showed better-aligned actin meshwork and displayed a spindle shape (Fig. 5d).

To further investigate the therapeutic mechanism, the production of intracellular ^1^O_2_ was traced by using DCFH-DA as a probe. A low-level ^1^O_2_ production was detected in the cells treated with Pt/BTNPs and light irradiation (Additional file 1: Figure S16), indicating the existence of limited photodynamic effect. The result was consistent with the cytotoxicity data (Fig. 5b). Such limited photodynamic effect of Pt/BTNPs also indicated the nanoparticles greatly hindered oxygen to get into the nanoassemblies [42, 43]. Taken together, the cellular cytotoxicity was mostly contributed by light-triggered PTX release rather than ^1^O_2_ production.

### In vivo anti-tumor efficacy

**Fig. 6:**
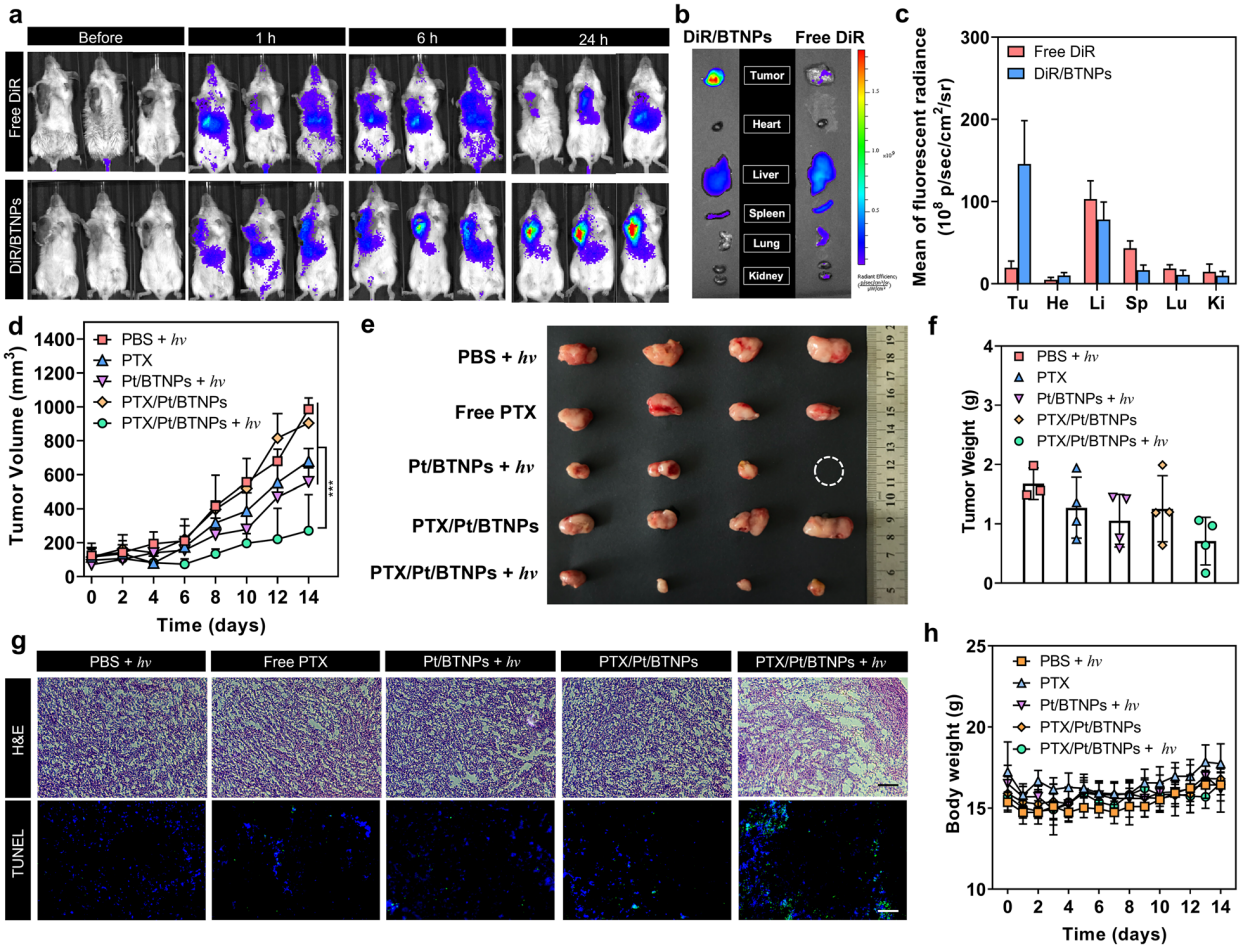
In vivo evaluation of biodistribution and therapeutic efficacy. **a** Representative IVIS fluorescence images of mice before and after intravenous injection of free DiR or DiR/ BTNPs at different time points (n = 3). **b** Ex vivo fluorescence imaging and (**c**) fluorescence radiance of tumor (Tu), heart (He), liver (Li), spleen (Sp), lung (Lu), and kidney (Ki) 24 h post-administrations (n = 3). Tumor volumes (**d**), photographs of the excised tumors after treatments (**e**), tumor weights (**f**) of mice over the 14-day treatments (n = 4). All the light irradiations were performed 24 h post-injection with a 635 nm laser (200 mW/cm^2^, 10 min). **g** H&E and TUNEL staining of the tumor slices of each group. Scale bar: 200 μm (H&E) and 50 μm (TUNEL). **h** Body weights of mice over the 14-day treatments (n = 4)

A breast cancer model with 4T1 tumor-bearing BALB/c mice was constructed for in vivo therapeutic efficacy evaluation. Since PTX is a commonly used first-line chemotherapeutics for breast cancer patients, the light-controlled therapeutic efficacy was evaluated in a mouse 4T1 breast tumor model [44]. To facilitate the visualization of Pt/BTNP biodistribution in vivo, a fluorescent dye, 1,1-dioctadecyl-3,3,3,3-tetramethylindotricarbocyanine iodide (DiR), was loaded in the nanoparticles (DiR/BTNPs) for in vivo imaging [45]. As shown in Fig. 6a, the mice intravenously injected with DiR/BTNPs showed apparent fluorescence signal at the tumor sites 1 h post-injection and the signal continuously increased within 24 h, indicating the accumulation of DiR/BTNPs in tumors. On the contrary, the mice injected with free DiR showed negligible fluorescence signal in tumors, which is due to the short blood circulation time and the rapid clearance of free DiR. At 24 h post-injection, the two groups of mice were sacrificed and the organs and tumors were excised for ex vivo fluorescence imaging (Fig. 6b, c). The results showed that DiR/BTNPs accumulated largely in tumors, but no obvious signal was observed for free DiR. The selective accumulation of the nanoparticles could be a result of passive targeting of the nanoparticles.

To evaluate the in vivo anti-tumor efficacy, 4T1 subcutaneous tumor-bearing mice were randomly divided into 5 groups (n = 4) when the tumor volume reached about 100 mm^3^. The 5 groups of mice were treated with PBS + *hv*, free PTX, Pt/BTNPs + *hv*, PTX/Pt/BTNPs, and PTX/Pt/BTNPs + *hv*, separately, with a dose of 5 mg/kg of body weight on PTX basis. Light irradiation was performed with a 635 nm laser (200 mW/cm^2^, 10 min) 24 h after the injection. The treatments were repeated every 3 days. Comparing to the control groups (PBS + *hv* and PTX/Pt/BTNPs), the mice treated with PTX/Pt/BTNPs plus light irradiation exhibited apparent inhibition of tumor growth within the 14-day treatment (Fig. 6d). Pt/BTNPs plus light irradiation showed slight suppression of tumor growth, which was comparable to free PTX treatment. The therapeutic effect of Pt/BTNPs with light irradiation may be attributed to the generated limited photodynamic effect. The tumors were excised after sacrificing the mice at the end of 14-day treatment. The size and weight of tumors in various groups were consistent with the result of tumor growth (Fig. 6e, f). Further evaluations were conducted by processing the tumors with hematoxylin and eosin (H&E) staining and terminal deoxynucleotidyl transferase dUTP nick-end labelling (TUNEL) staining (Fig. 6g). Notable necrosis and apoptosis occurred only in the tumors treated with PTX/Pt/BTNPs + *hv*. Additionally, no obvious body weight change in mice was found with the treatments of the studied formulations (Fig. 6h), suggesting their desirable biocompatibility at the used concentration. The H&E staining of organ slices, including heart, lung, liver, spleen and kidney, displayed no apparent abnormality after the treatments, showing that no organ damage occurred during the treatments (Additional file 1: Figure S17). These results proved that the red light-triggerable drug release system is a safe formulation with significant anti-tumor efficacy after red-light irradiation in vivo.

## Conclusions

In this study, one-photon upconversion-like photolysis process was firstly used for red light-triggered nanoparticle disassembly and drug release. The photocleavable BODIPY-derived trigonal molecule, BTAEA, could self-assemble into nanoparticles that can enclose various cargos including dyes and drugs. While loading PtTPBP as the photosensitizer, this system was able to respond to red light at 635 nm, resulting in BTAEA photolysis, dissociation of the nanoparticles and cargo release. Such nanostructure can prevent oxygen quenching during energy-transfer processes to facilitate photolysis of photocleavable molecules. Upon red-light irradiation, the nanoparticles dissociated and displayed effective drug release performance, which led to excellent anti-tumor efficacy both in vitro and in vivo. In all, this study provides a versatile platform for tumor-specific cargo release, highlighting the convenience and efficiency of applying energy transfer-based photolysis in drug delivery. The simple molecular self-assembly and disassembly strategy overcomes the limitations of both oxygen quenching in photolysis process and non-responsiveness of the traditional nanocarriers. For the easily accessible tumors, such as ocular tumors, skin tumors and breast tumors, in which light can readily reach the lesion and trigger drug release, photoresponsive drug delivery systems can be used and activated directly. Moreover, for the tumors in deeper area, light delivery devices, for example, optical fibers, can be applied to deliver light and activate photoresponsive systems. In all, this study advances the development of long-wavelength light-responsive drug delivery systems for cancer therapy.

## Methods

### Materials

Diethyl ester, *N*,*N*-diisopropylethylamine (DIPEA), tri-(2-aminoethyl) amine (TAEA), sodium sulphate anhydrous (Na_2_SO_4_), 4-(dimethylamino) pyridine (DMAP), *N*,*N*-dimethylformamide (DMF), dimethyl sulfoxide (DMSO) were obtained from Sigma-Aldrich (Darmstadt, Germany). 4-Nitrophenyl chloroformate was obtained from Alfa Aesar (Thermo Fisher, Heysham, Lancashire, UK). Pyridine, anhydrous dichloromethane, triethylamine (TEA), hydrochloric acid (HCl), 3-(4,5-dimethyl-2-thiazolyl)-2,5-diphenyl-2-*H*-tetrazolium bromide (MTT) and other unlisted chemicals were obtained from J&K Co., Ltd (Beijing, China). Acetonitrile (ACN), methanol, hexene, ethyl acetate, tetrahydrofuran and other solvents were obtained from Oriental Co., Ltd (Hong Kong, China). DSPE-PEG_2000_ was supplied by Ponsure Biological Co., Ltd (Shanghai, China).

### Synthesis of (BODIPY)_3_-TAEA (BTAEA)

The synthesis of BTAEA was descripted in Additional file 1: Figure S1. Compound 1–3 were synthesized according to the reported methods in our previous study without any modification [20].

Compound 4 (BODIPY-4-NPC): Compound 3 (124 mg, 0.5 mmol) was dissolved in dry tetrahydrofuran (5 mL) in a flask and protected by nitrogen gas in the dark. Then DIPEA (220 µL, 5 eq) was added into the solution. The mixture was cooled to 0 °C and stirred for 10 min. 4-Nitrophenyl chloroformate (4-NPC) (217.7 mg, 4 eq) dissolved in THF was added slowly. Then pyridine (5.5 µL, 0.25 eq) was added. The mixture was stirred for 150 min at room temperature. The organic layer was concentrated under vacuum and the residue was purified with a chromatography column by using 1:1 hexene/DCM (v/v) to give compound 4 as orange powder. The structure and purity of the product were confirmed by ^1^H-NMR spectroscopy.

Compound 5 (iodine substituted BODIPY-4-NPC): Compound 4 (80 mg, 0.2 mmol) and zinc oxide powder (60 mg, 3.6 eq) were dissolved in dry THF (3 mL) and protected by nitrogen gas in the dark. The mixture was then immersed into ice bath. ICl (100 mg, 3 eq) was dissolved in THF and then added into the mixture drop by drop. The solvent was removed after 15 min of reaction and the residue was purified by silica column eluted by hexene/DCM (1.5:1) to give purple-red powder as the product. The structure and purity of the product were confirmed by ^1^H-NMR spectroscopy.

Compound 6 (BODIPY_3_-TAEA, BTAEA): Compound 5 (40 mg, 0.1 mmol) was dissolved in 1.5 mL dry DCM under nitrogen gas and cooled to 0 °C. DIPEA (35 μL, 0.2 mmol) was added and stirred for 15 min. The solution of TAEA (5 μL, 0.03 mmol) in 1 mL dry DCM was slowly added into the above mixture at 0 °C. The mixture was then warmed to room temperature. After stirring for about 1 h, more DIPEA (35 μL) was added. The mixture was continuously stirred for 48 h. Thin-layer chromatography (TLC) was used to confirm the complete consumption of compound 5. DCM/MeOH (20:1) was used to purify the final product with a silica column as purple-red powder. The structure and purity of the product were confirmed by ^1^H-NMR and ESI–MS spectra.

### Fabrication and characterization of BTAEA nanoassemblies

Flash nanoprecipitation method was used to fabricate the nanoassemblies following the reported method [25, 27]. Briefly, BTAEA (10 mg/mL, 5 μL) and DSPE-mPEG_2000_ (20 mg/mL, 1 μL) were dissolved in DMSO separately and then mixed to form a stock solution. The stock solution was added into 200 μL of filtrated water with vortexing to form BTAEA nanoassemblies (BTNPs). The resulted solution of nanoparticles containing organic solvent can be purified via centrifugation with ultrahigh-speed low-temperature centrifuge (ST 8R, Thermo Fisher Scientific, Waltham, MA, USA). The solution was centrifuged at 3000×*g* for 10 min, which was repeated for at least three times until no precipitate was observed. The supernatant was then collected and further centrifuged at 30,000×*g* for 20 min. The nanoparticles were obtained as the precipitate located at the bottom of the tube. The precipitate was resuspended in water or PBS. The concentration of BTAEA was then determined by HPLC. TEM images, mapping images and EDX spectrum was recorded by Philips CM100 transmission electron microscope. Size distribution and zeta potential of the nanoassemblies were measured by dynamic light scattering instrument (ZS90, Malven Instrument, southborough, MA, USA). To test the serum stability of BTNPs, BTNPs were prepared and adjusted to 2 mg/mL in aqueous solution. The BTNPs solution was then diluted by concentrated FBS (2×) to a final concentration of 1 mg/mL. The absorbance at 560 nm was measured at different time points (0 h, 4 h, 12 h and 24 h).

To prepare Pt/BTNPs, PtTPBP was added to the stock solution and the same procedures were used. To prepare PTX/Pt/BTNPs, PtTPBP and PTX were added to the stock solution and the same procedures were used. The concentrations of BTAEA and PTX were determined by HPLC analysis. Loading capacity and encapsulation efficiency were calculated as follow:

$${\mathrm{Loading\,capacity}}\left({\%}\right)=\frac{\mathrm{weight\,of\,the\,payload}}{\mathrm{weight\,of\,the\,nanoparticles }}\times 100{\%}.$$

$${\mathrm{Encapsulation\,efficiency }}\left({\%}\right)=\frac{\mathrm{weight\,of\,the\,loaded\,payload}}{\mathrm{weight\,of\,the\,feeded\,payload}}\times 100{\%}.$$

### TD-DFT calculations

Time-dependent density-functional theory (TD-DFT) was used to calculate the energy levels of the S_1_ states and T_1_ states of BTAEA and PtTPBP. The calculation procedures were performed based on the Gaussian 16 software package. Geometry optimizations were calculated at the B3LYP/6-31G(d) (LANL2DZ on I) level.

### Phosphorescence quenching by TTET

The TTET process was verified by determining the phosphorescence of PtTPBP that can be quenched by BTAEA. The experiments were conducted according to the previous study [20]. Briefly, the phosphorescence of PtTPBP (10^–5^ M) was recorded in the presence of different concentrations of BTAEA (0, 5 × 10^–7^ M, 2 × 10^–6^ M, 4 × 10^–6^ M, 7 × 10^–6^ M, and 10^–5^ M) in the mixed solvent of 90% methanol and 10% toluene. The solution was N_2_-saturated by purging N_2_ for 10 min to avoid oxygen quenching. Moreover, the quenching constants (k_q_) were calculated according to the Stern–Volmer Eq. (1).

1 $$ \frac{{\text{I}}{0}}{\text{It}}{=}{\text{1+}} \,{\text{k}\small{q}}\left[{\text{Q}}\right],$$

(I_0_: phosphorescence intensity of PtTPBP in solution; I_t_: phosphorescence intensity of PtTPBP in the presence of BTAEA; [Q]: concentration of BTAEA).

The rate of TTET process was quantified as *k*_*TTET*_, which can be calculated based on the below Eq. (2).

2 $${\text{k}{\small{{TTET}}}}{=}\frac{{\text{k}}{\text{q}}}{ \, {\tau}{0}},$$

($\tau$_0_: phosphorescence lifetime of PtTPBP without quencher; According to the literature, $\tau$_0_ = 42.92 μs [20]).

### Quantitative analysis of BTAEA photolysis

To quantitatively determine the photolysis of BTAEA, the concentrations of BTAEA and released BODIPY were measured by HPLC. The aqueous solution of PtTPBP-loaded BTNPs (Pt/BTNPs) or plain BTNPs (50 µL, 1 mg/mL, on BTAEA basis) was irradiated by 635 nm laser (50 mW/cm^2^) (LWRL635, Laserwave, Beijing, China) or 530 nm laser (50 mW/cm^2^) (LWRL530, Laserwave, Beijing, China) for different time periods (0, 1, 3, 5, and 10 min). The irradiated solution was mixed with 50 µL acetonitrile to dissolve the nanoassemblies. The solution was analyzed by HPLC (Angilent 1260 Infinity, CA, USA), and the photolysis profile was recorded based on the irradiation time and the determined concentrations.

### Characterization of light-triggered payload release

Nile red-loaded nanoassemblies, NR/BTNPs and NR/Pt/BTNPs, were prepared by the above method. The solutions were put into a cuvette and irradiated by light (635 nm, 50 mW/cm^2^, 0–10 min). At different time points (0, 1, 2, 3, 5 and 10 min), the fluorescence was measured by a spectrometer (SpectraMax^®^ M4, Molecular Devices LLC, San Jose, CA, USA) without any dilution.

### Cell culture

Mouse breast cancer 4T1 cells were purchased from Stem Cell Bank, Chinese Academy of Sciences. Cells were cultured in DMEM (Gibco) supplemented with 10% FBS (Gibco) and 100 units/mL antibiotics (Penicillin–Streptomycin, Gibco) at 37 °C in a 5% CO_2_ humidified atmosphere.

### Cellular uptake analysis

For confocal imaging, 4T1 cells were plated in the confocal plates (Corning, 200350, Cell Culture-Treated) at a density of 5000 cells/well. Different formulations including PBS, NR-labelled BTNPs and Pt/BTNPs (5 μg/mL, on BTAEA basis) were added into the medium. Then the lysosomes were labelled with Lysotracker® Green (Thermo, Heysham, Lancashire, UK). For the light irradiated group, red light (50 mW/cm^2^) was applied at the bottom of the cell plate for 10 min. After 4 h incubation, the medium was removed and replaced by fresh medium after washing the cells with PBS for 3 times. The cells were observed under a confocal laser scanning microscope (LSM 980, Carl Zeiss, Germany).

### Cytotoxicity analysis

Cell viabilities were determined by MTT assay. Briefly, 4T1 cells were cultured on 96-well plates at a primary density of 5000 cells/well and incubated for 24 h before adding the formulations. The medium was replaced with formulation-containing medium at different concentrations. After 4 h incubation, the cells were washed with PBS for 3 times and the light irradiation (635 nm, 50 mW/cm^2^) was performed. MTT solution (10 μL/well) was added after 24 h of incubation and the OD490, OD570 and OD630 values were measured for the calculation of cell viability. The fluorescence imaging of microtubule was conducted by staining actin with Alexa Fluor 488-phallodin (Thermo Fisher, CA, USA) in 1% BSA-containing PBS. The cells treated with PBS, PTX or PTX/Pt/BTNPs were incubated for 24 h and washed with PBS for 3 times. The CLSM images were collected thereafter.

### Intracellular singlet oxygen detection

Singlet oxygen generation was measured in cells by 2′-7′dichlorofluorescin diacetate (DCFH-DA) as an indicator. 4T1 cells were seeded in confocal dishes (Corning, 200350, Cell Culture-Treated) at a density of 5000 cells/well and treated with PBS, BTNPs, Pt/BTNPs or PTX/Pt/BTNPs for 4 h. Subsequently, the cells were washed with PBS for 3 times and incubated with DCFH-DA (10 μM) for 30 min. The cells were irradiated (635 nm, 50 mW/cm^2^, 10 min) thereafter. The intracellular fluorescence was observed by CLSM imaging to evaluate the intracellular ^1^O_2_ generation.

### Animals

BALB/c mice (female, 4 weeks, about 15 g) were purchased from the Animal Center of Qinglong Mountain (Nanjing, China). The animal experiments were conducted according to protocol that was approved by the ethics committee of China Pharmaceutical University.

### In vivo biodistribution

For the subcutaneous tumor model, mice were injected with 2 × 10^7^ 4T1 cells subcutaneously. The mice were then further kept in SPF condition for 5–7 days until tumors were observed and reached at about 100 mm^3^. Mice were treated with free DiR or DiR/BTNPs via intravenous injection with a dose at 100 μg/kg. Fluorescence imaging was performed at 0, 1, 6, and 24 h post injection. At 24 h, mice were euthanized and the tumors and major organs (heart, liver, spleen, lung, kidney) were excised for ex vivo imaging.

### Anti-tumor effects in 4T1 tumor-bearing mice

The antitumor efficacy of the formulations in the presence or absence of light was investigated with 4T1 tumor-bearing mouse model. The mice were randomly divided into 5 groups (n = 4) when the tumor size reached about 100 mm^3^. Different formulations were intravenously administrated: (1) PBS + *hv*; (2) PTX; (3) Pt/BTNPs; (4) PTX/Pt/BTNPs; (5) PTX/Pt/BTNPs + *hv*. For the irradiation groups, light irradiation (635 nm, 200 mW/cm^2^, 10 min) was performed 24 h post injection. The dose for each treatment was set as 5 mg/kg on PTX basis. The treatments were repeated every three days. Tumor sizes and body weights were measured during the period, and the tumor volume was calculated as V = 1/2 × width^2^ × length. On Day 14, all of the mice were euthanized, and the tumors and major organs were excised and sliced for H&E staining and immunohistochemical analysis.

### Statistical analysis

All experiments were conducted three times or more independently (n ≥ 3). Data were presented as the mean ± standard deviation (SD). The one-way ANOVA-LSD and Independent-Samples t-test were adopted to determine the statistical significance of differences by Graphpad Prism 8.0 software.

## Supplementary Information

**Additional file 1:** **Figure S1**. Synthesis of the BTAEA molecule. **Figure S2**. ^1^H-NMR spectrum of Compound 4. **Figure S3**. ESI-MS spectrum of Compound 4. **Figure S4**. ^1^H-NMR spectrum of Compound 5. **Figure S5**. ESI-MS spectrum of Compound 5. **Figure S6**. ^1^H-NMR spectrum of Compound 6 (BTAEA). **Figure S7**. ESI-MS spectrum of Compound 6 (BTAEA). **Figure S8**. Serum stability of PTX/Pt/BTNPs for 24 h under 37 ^o^C. **Figure S9**. Standard curves of BTAEA (a) and PTX (b) based on areas of HPLC elution peaks. **Figure S10**. (a) Size and (b) PDI of the BTAEA nanoparticles contained different ratios (5%, 10% and 20%) of DSPE-mPEG. (c) Encapsulation efficiency and loading capacity of PTX in BTNPs with different feeding ratios (PTX/BTAEA, w/w). **Table S1**. Composition of PTX/Pt/BTNPs. **Figure S11**. Photolysis of BTAEA in Pt/BTNPs with 635 nm light irradiation (50 mW/cm^2^). **Figure S12**. LC-MS spectrum of an irradiated solution of Pt/BTNPs. **Figure S13**. Photolysis of BTAEA in BTNPs with 635 nm light irradiation (50 mW/cm^2^). **Figure S14**. Photolysis of BTAEA in BTNPs with 530 nm light irradiation (50 mW/cm^2^). **Figure S15**. TEM image and DLS data of PTX/Pt/BTNPs in aqueous solutions after red light irradiation (635 nm, 50 mW/cm^2^, 10 min). **Figure S16**. CLSM images of intracellular ROS generation in 4T1 cells treated with PBS, BTNPs, Pt/BTNPs and PTX/Pt/BTNPs, separately. **Figure S17**. Representative photomicrographs of hematoxylin & eosin-stained sections of heart, lung, spleen, liver and kidney from different treatment groups.

## Data Availability

All data generated or analyzed during this study are included in this published article and the Additional Information.

## Abbreviations

BTAEA: (BODIPY)_3_-tris(2-aminoethyl) amine
PtTPBP: Platinum (II) tetraphenyltetrabenzoporphyrin
PTX: Paclitaxel
DSPE-mPEG_2000_: 1,2-Distearoyl-sn-glycero-3-phosphoethanolamine-*N*-[methoxy(polyethyleneglycol)-2000]
TTET: Triplet–triplet energy transfer
S_0_: Singlet ground state
S_1_: The first singlet excited state
T_1_: The first triplet excited state
