# Supplementary material for: One-photon red light-triggered disassembly of small-molecule nanoparticles for drug delivery
Source: J Nanobiotechnology. 2021 Nov 4;19:357. doi: 10.1186/s12951-021-01103-z (PMC8567723; doi:10.1186/s12951-021-01103-z)
Supplement: Supplementary file 1 — Additional file 1: Figure S1. Synthesis of the BTAEA molecule. Figure S2. 1H-NMR spectrum of Compound 4. Figure S3. ESI-MS spectrum of Compound 4. Figure S4. 1H-NMR spectrum of Compound 5. Figure S5. ESI-MS spectrum of Compound 5. Figure S6. 1H-NMR spectrum of Compound 6 (BTAEA). Figure S7. ESI-MS spectrum of Compound 6 (BTAEA). Figure S8. Serum stability of PTX/Pt/BTNPs for 24 h under 37 oC. Figure S9. Standard curves of BTAEA (a) and PTX (b) based on areas of HPLC elution peaks. Figure S10. (a) Size and (b) PDI of the BTAEA nanoparticles contained different ratios (5%, 10% and 20%) of DSPE-mPEG. (c) Encapsulation efficiency and loading capacity of PTX in BTNPs with different feeding ratios (PTX/BTAEA, w/w). Table S1. Composition of PTX/Pt/BTNPs. Figure S11. Photolysis of BTAEA in Pt/BTNPs with 635 nm light irradiation (50 mW/cm2). Figure S12. LC-MS spectrum of an irradiated solution of Pt/BTNPs. Figure S13. Photolysis of BTAEA in BTNPs with 635 nm light irradiation (50 mW/cm2). Figure S14. Photolysis of BTAEA in BTNPs with 530 nm light irradiation (50 mW/cm2). Figure S15. TEM image and DLS data of PTX/Pt/BTNPs in aqueous solutions after red light irradiation (635 nm, 50 mW/cm2, 10 min). Figure S16. CLSM images of intracellular ROS generation in 4T1 cells treated with PBS, BTNPs, Pt/BTNPs and PTX/Pt/BTNPs, separately. Figure S17. Representative photomicrographs of hematoxylin & eosin-stained sections of heart, lung, spleen, liver and kidney from different treatment groups. [file 12951_2021_1103_MOESM1_ESM.docx]

Supporting Information

**One-photon Red Light-triggered Disassembly of Small-molecule Nanoparticles for Drug Delivery**

*Kaiqi Long,^abc^ Han Han,^cd^ Weirong Kang,^abc^ Wen Lv,^c^ Lang Wang,^e^ Yufeng Wang,^e^ Liang Ge*,^d^ Weiping Wang*,^abc^*

a. State Key Laboratory of Pharmaceutical Biotechnology, The University of Hong Kong, Pokfulam, Hong Kong, China.

b. Department of Pharmacology & Pharmacy, Li Ka Shing Faculty of Medicine, The University of Hong Kong, Pokfulam, Hong Kong, China.

c. Dr Li Dak-Sum Research Centre, The University of Hong Kong, Pokfulam, Hong Kong, China

d. State Key Laboratory of Natural Medicines and Department of Pharmaceutics, China Pharmaceutical University, Nanjing, Jiangsu, China.

e. Department of Chemistry, The University of Hong Kong, Pokfulam, Hong Kong, China.

Prof. Liang Ge, Email: geliang1981@hotmail.com

Dr. Weiping Wang, Email: wangwp@hku.hk

**Content**

1. Fig. S1-S17..........................................................................................p3-12; p14-20
2. Table S1................................................................................................................p13


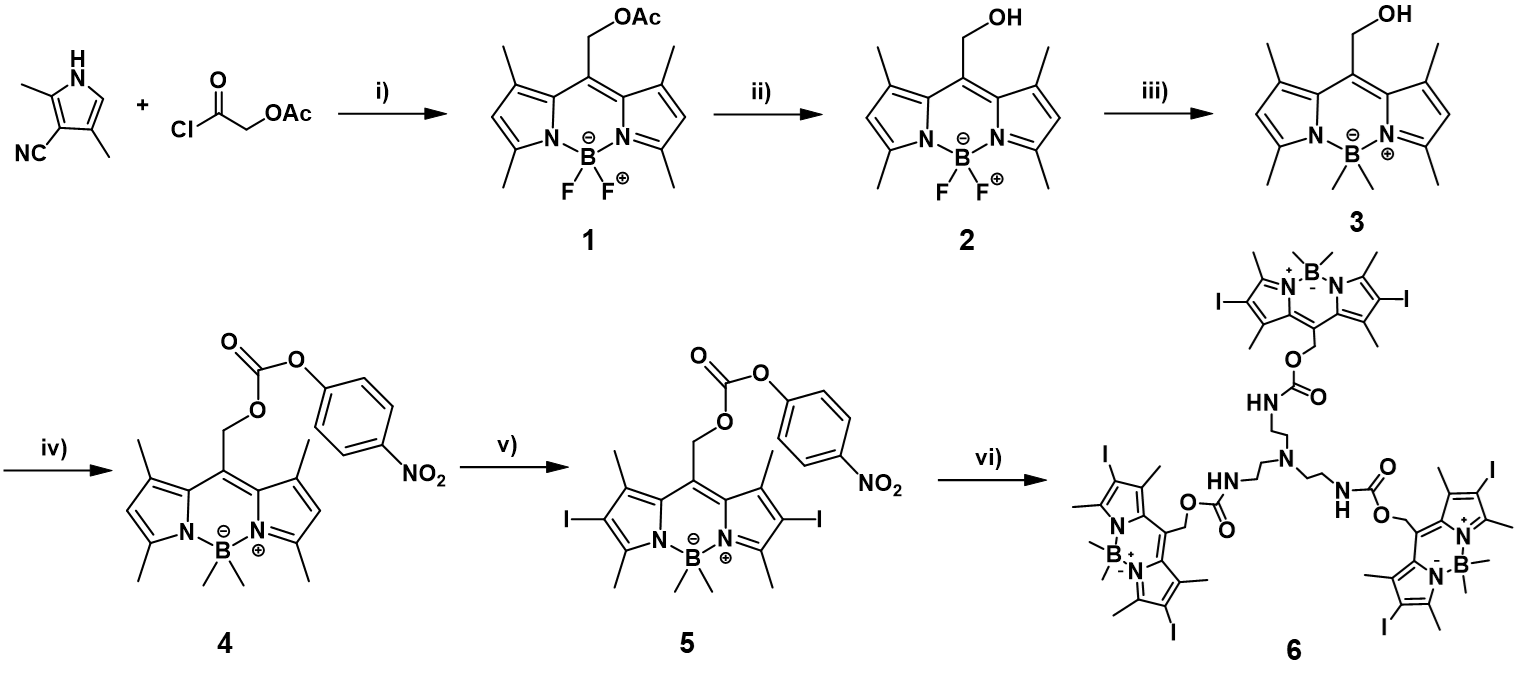


**Figure S1.**  Synthesis of the BTAEA molecule. The brief reaction conditions: i) DIPEA, BF_3_-OEt_2_, DCM, 40 ^o^C, 1 h, then r.t. overnight; ii) NaOH, DCM/MeOH, r.t., 4h; iii) MgMeI, Et_2_O, r.t., 3h; iv) 4-nitrophenyl chloroformate, DIPEA, DCM, pyridine, THF, r.t., overnight; v) ZnO, ICl, THF, 0 ^o^C, 15 min; vi) TAEA, DIPEA, DCM, r.t., 24 h.


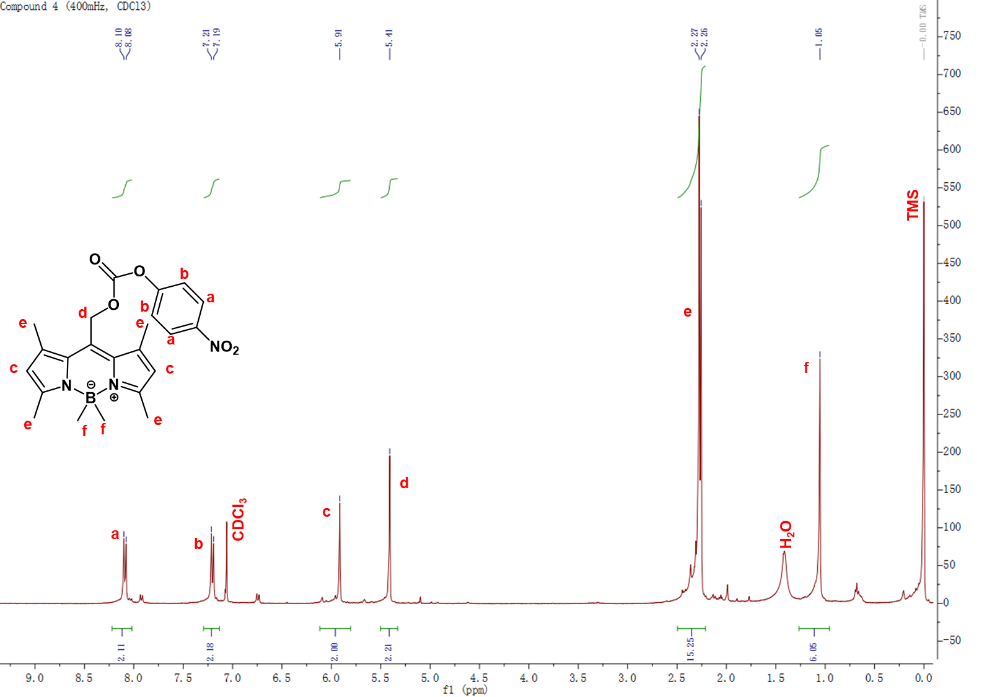


**Figure S2.** ^1^H-NMR spectrum of Compound 4.


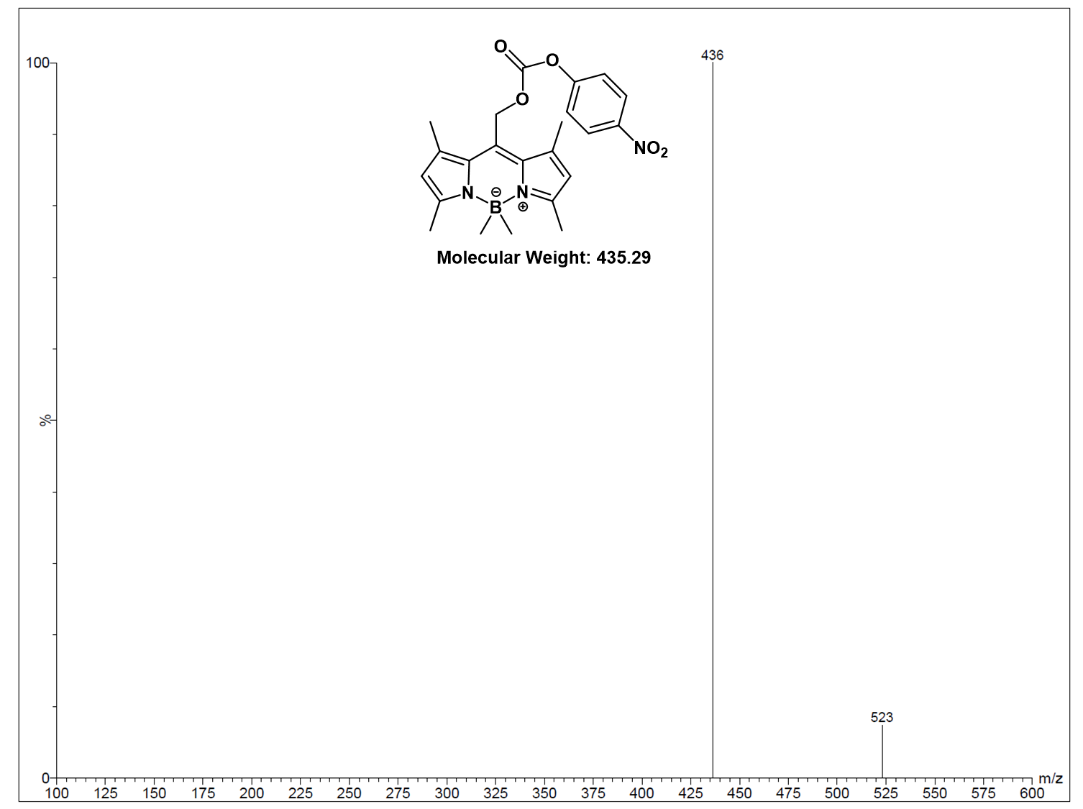


**Figure S3.** ESI-MS spectrum of Compound 4.


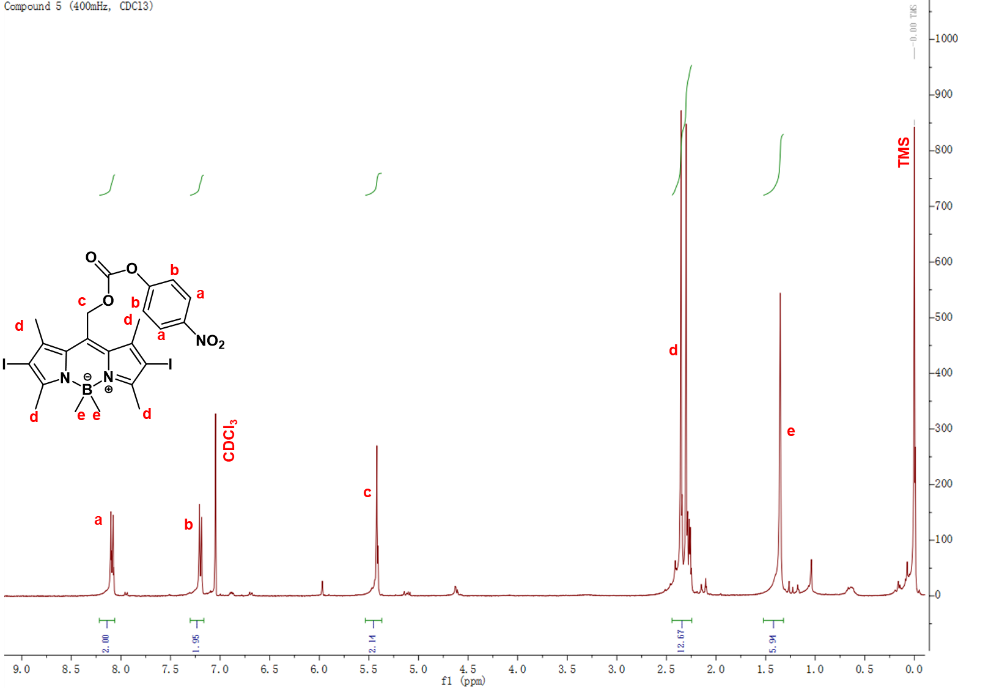


**Figure S4.** ^1^H-NMR spectrum of Compound 5.


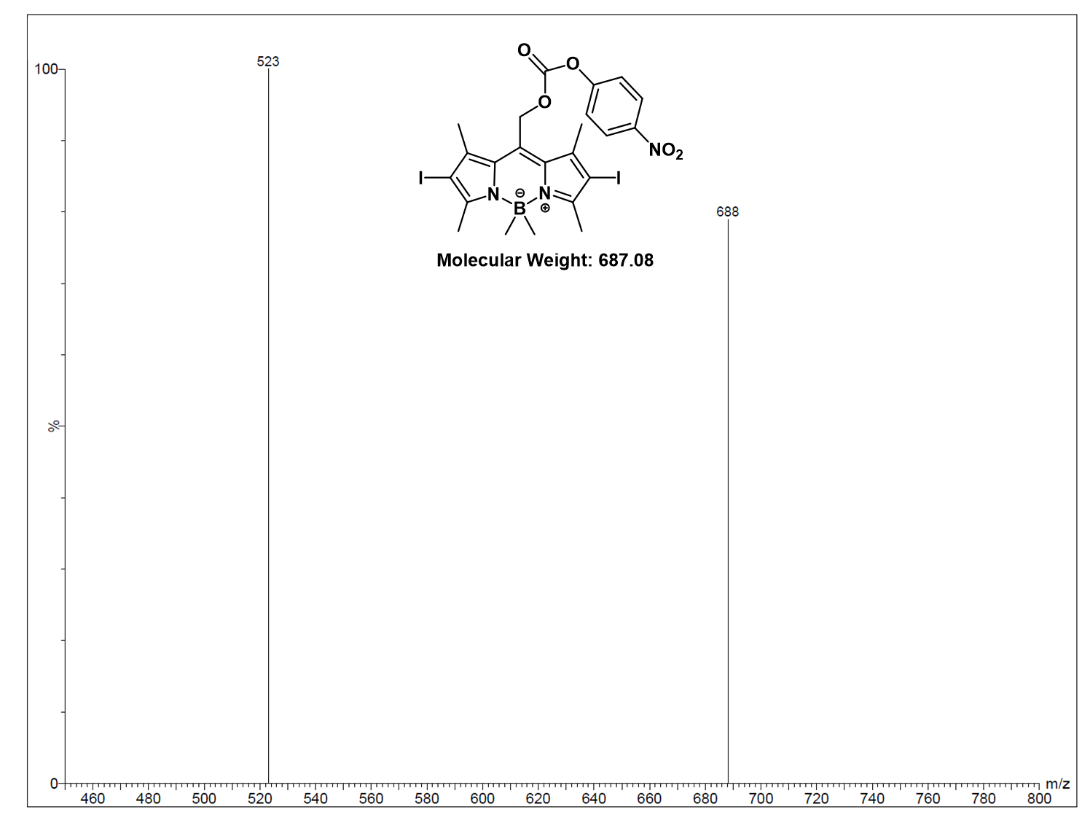


**Figure S5.** ESI-MS spectrum of Compound 5.


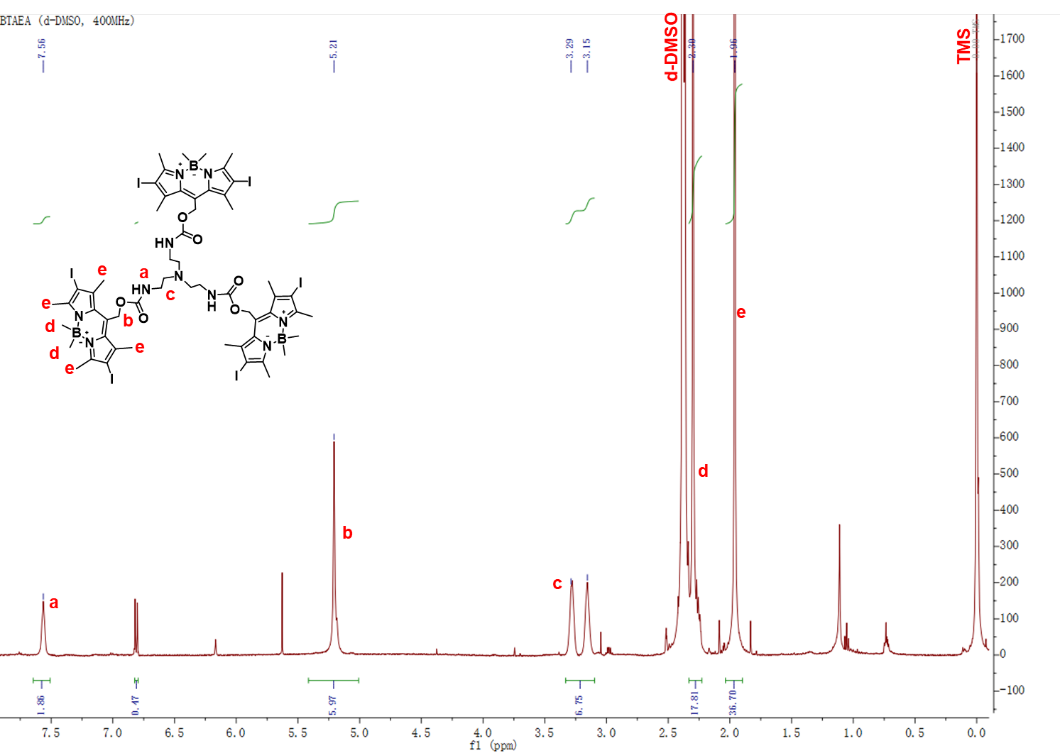


**Figure S6.** ^1^H-NMR spectrum of Compound 6 (BTAEA).


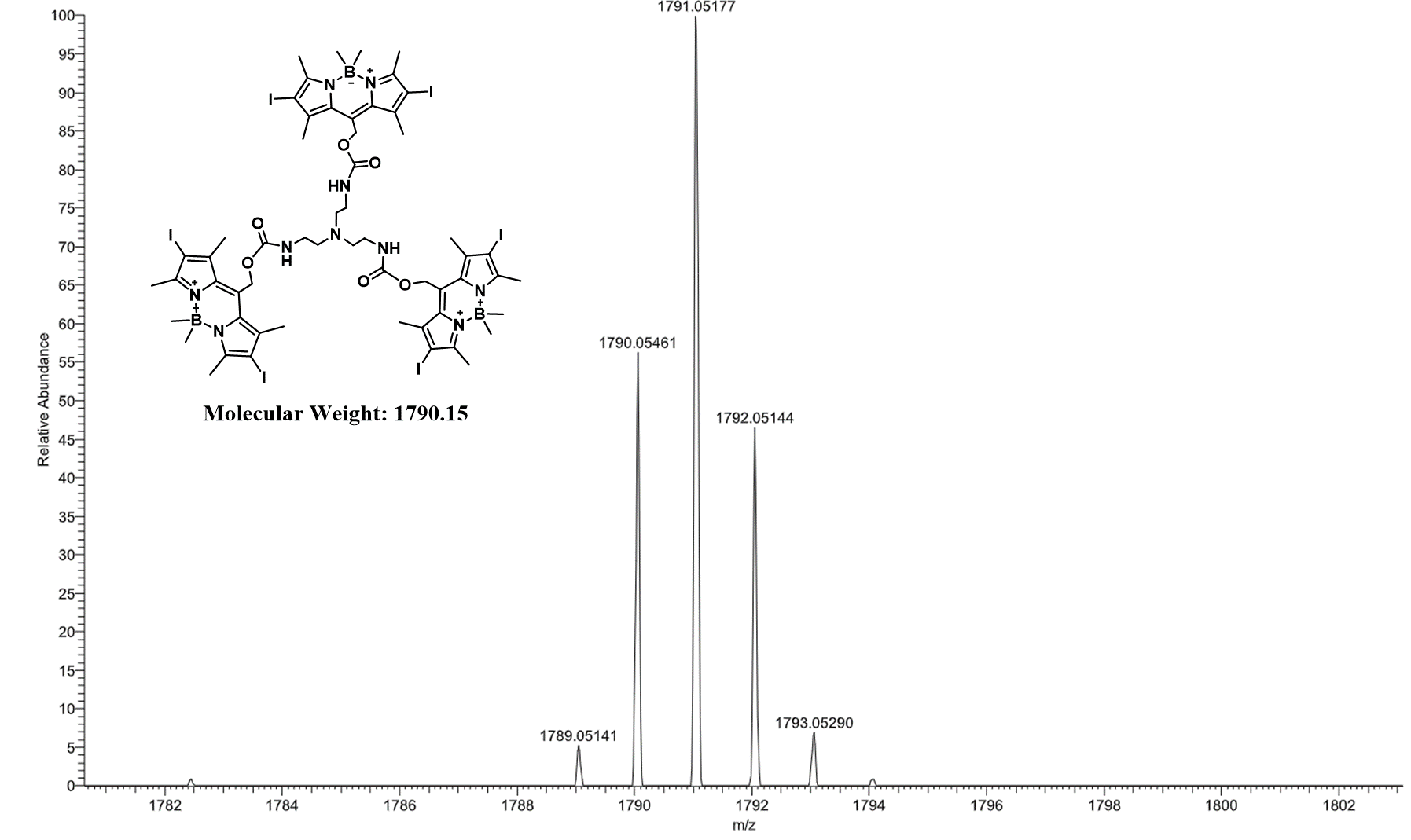


**Figure S7.** ESI-MS spectrum of Compound 6 (BTAEA).


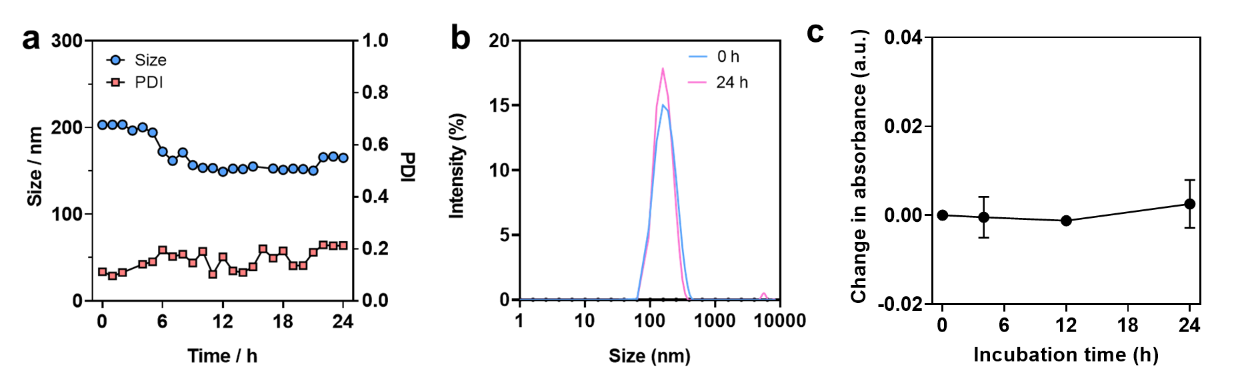


**Figure S8.** Serum stability of PTX/Pt/BTNPs for 24 h under 37 ^o^C. (a) Size and PDI values, (b) Size distribution curves at 0 h and 24 h of PTX/Pt/BTNPs incubated in FBS-contained DMEM medium. (c) Change of the absorbance at 560 nm of PTX/Pt/BTNPs incubated in 100% for 24 h. (n = 3)


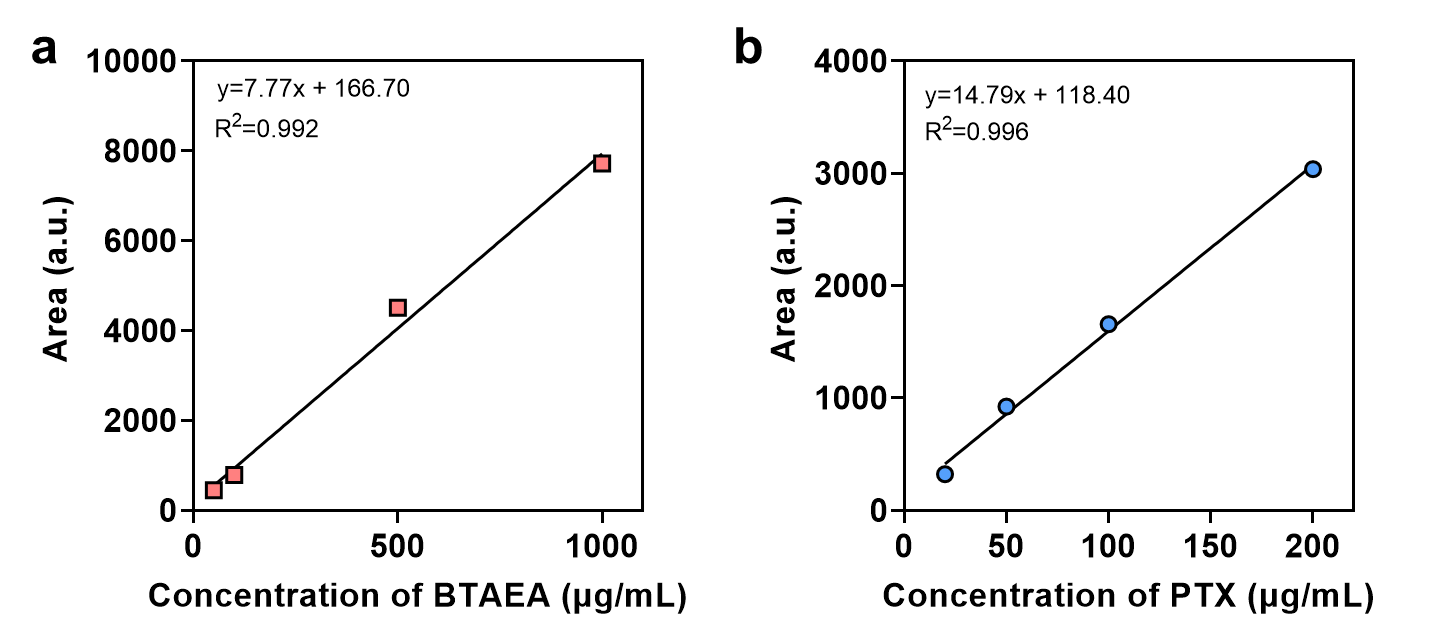


**Figure S9.** Standard curves of BTAEA (a) and PTX (b) based on areas of HPLC elution peaks (n = 3).


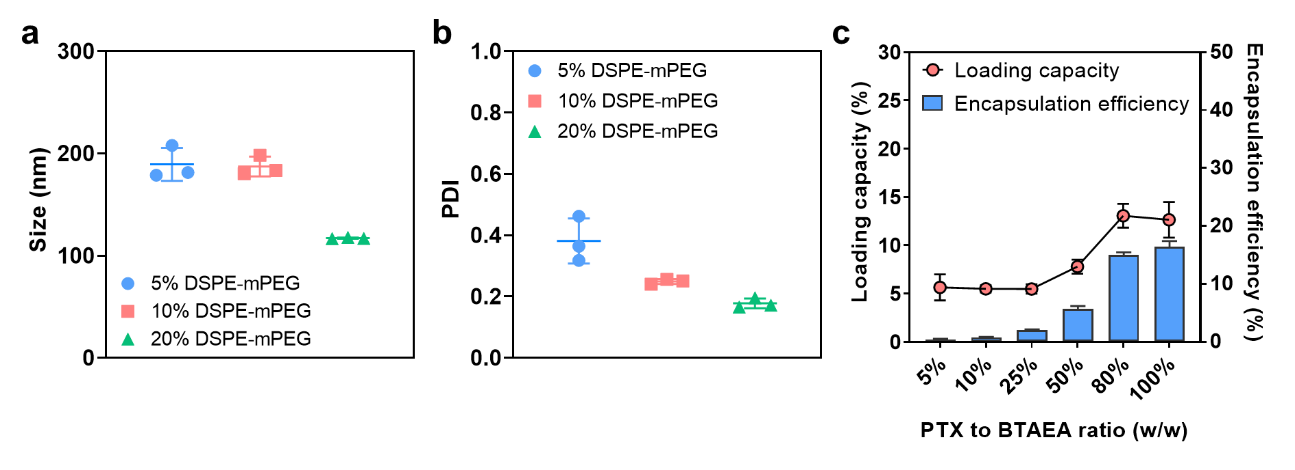


**Figure S10.** (a) Size and (b) PDI of the BTAEA nanoparticles contained different ratios (5%, 10% and 20%) of DSPE-mPEG (n=3). (c) Encapsulation efficiency and loading capacity of PTX in BTNPs with different feeding ratios (PTX/BTAEA, w/w) (n = 3).

**Table S1.** Composition of PTX/Pt/BTNPs


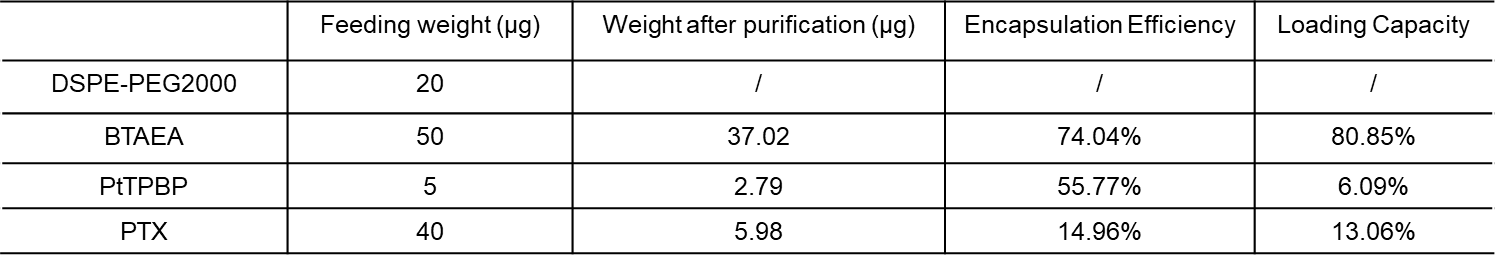


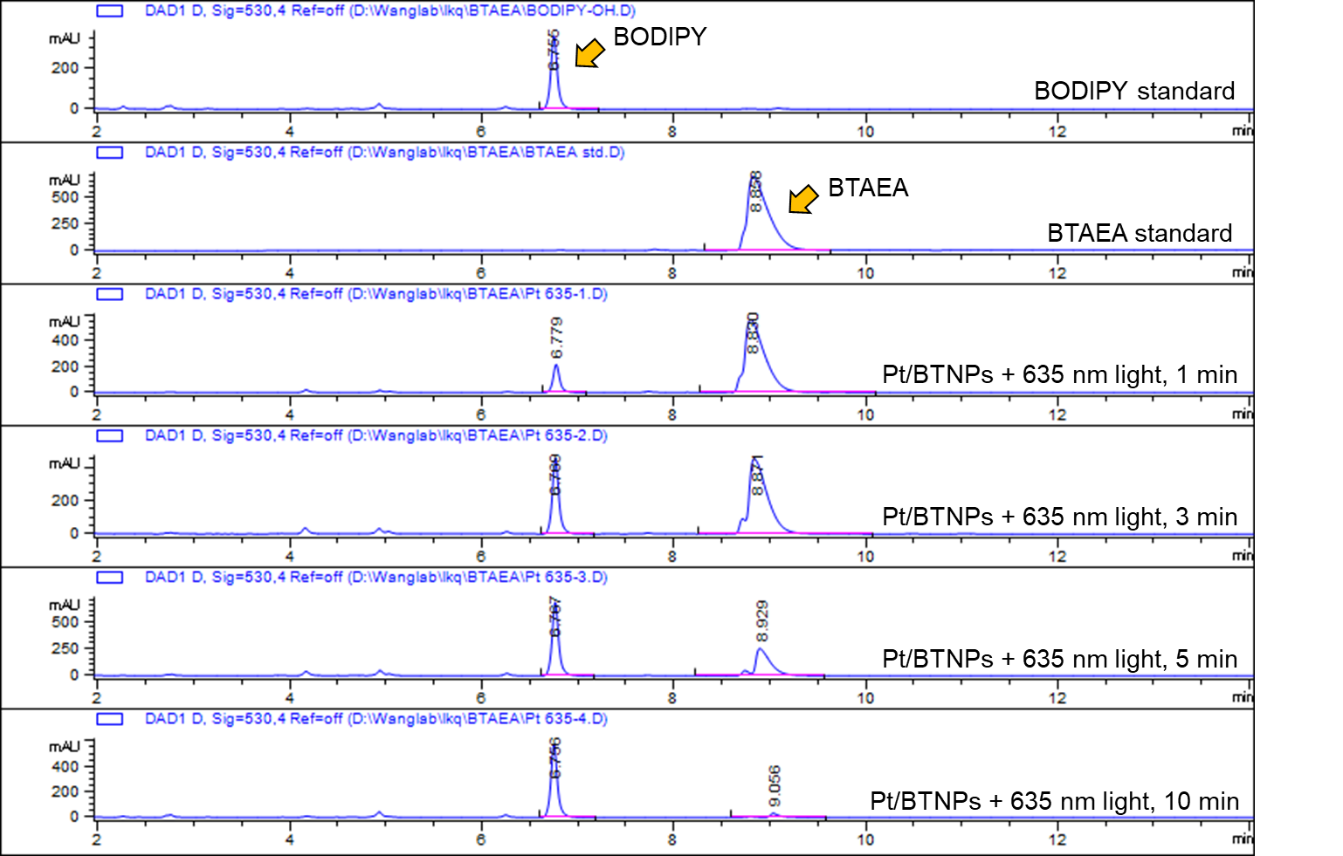


**Figure S11.** Photolysis of BTAEA in Pt/BTNPs with 635 nm light irradiation (50 mW/cm^2^).


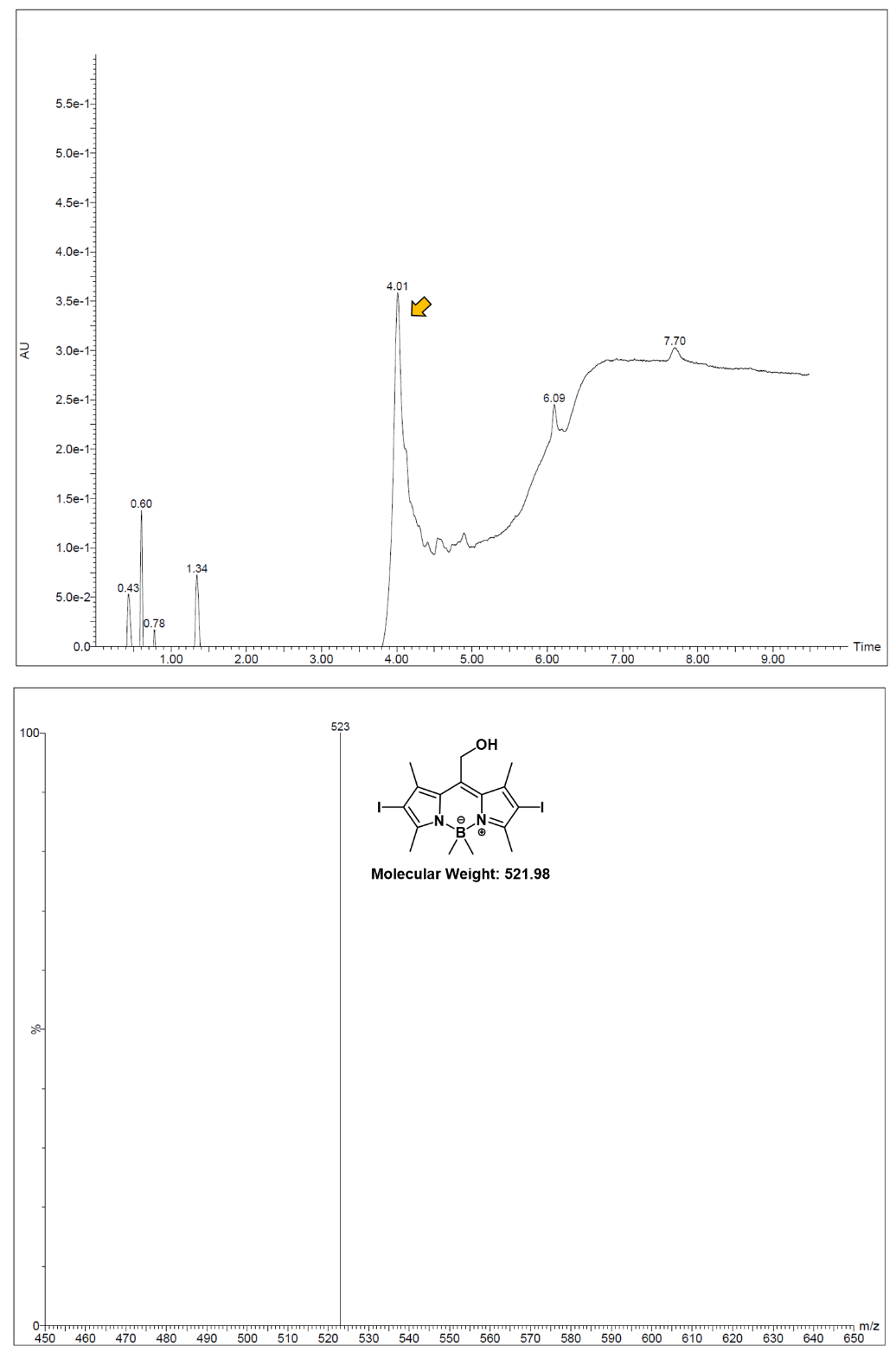


**Figure S12.** LC-MS spectrum of an irradiated solution of Pt/BTNPs (mode: positive ion). The solution of Pt/BTNPs was irradiated by 635 nm light (50 mW/cm^2^) for 10 min. The MS spectrum of the elution peak at 4.01 min showed the peak of the proposed photocleavage product, BODIPY.


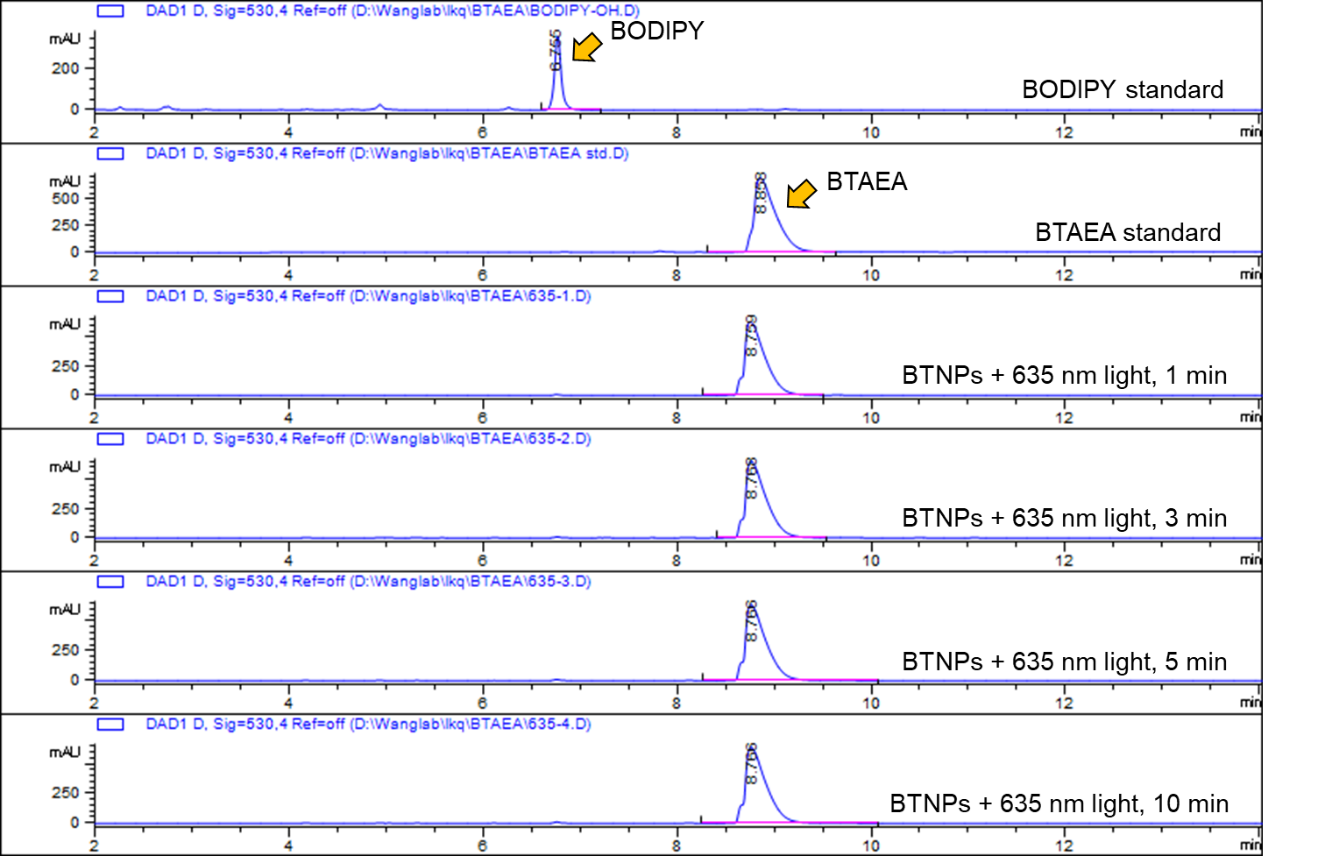


**Figure S13.** Photolysis of BTAEA in BTNPs with 635 nm light irradiation (50 mW/cm^2^).


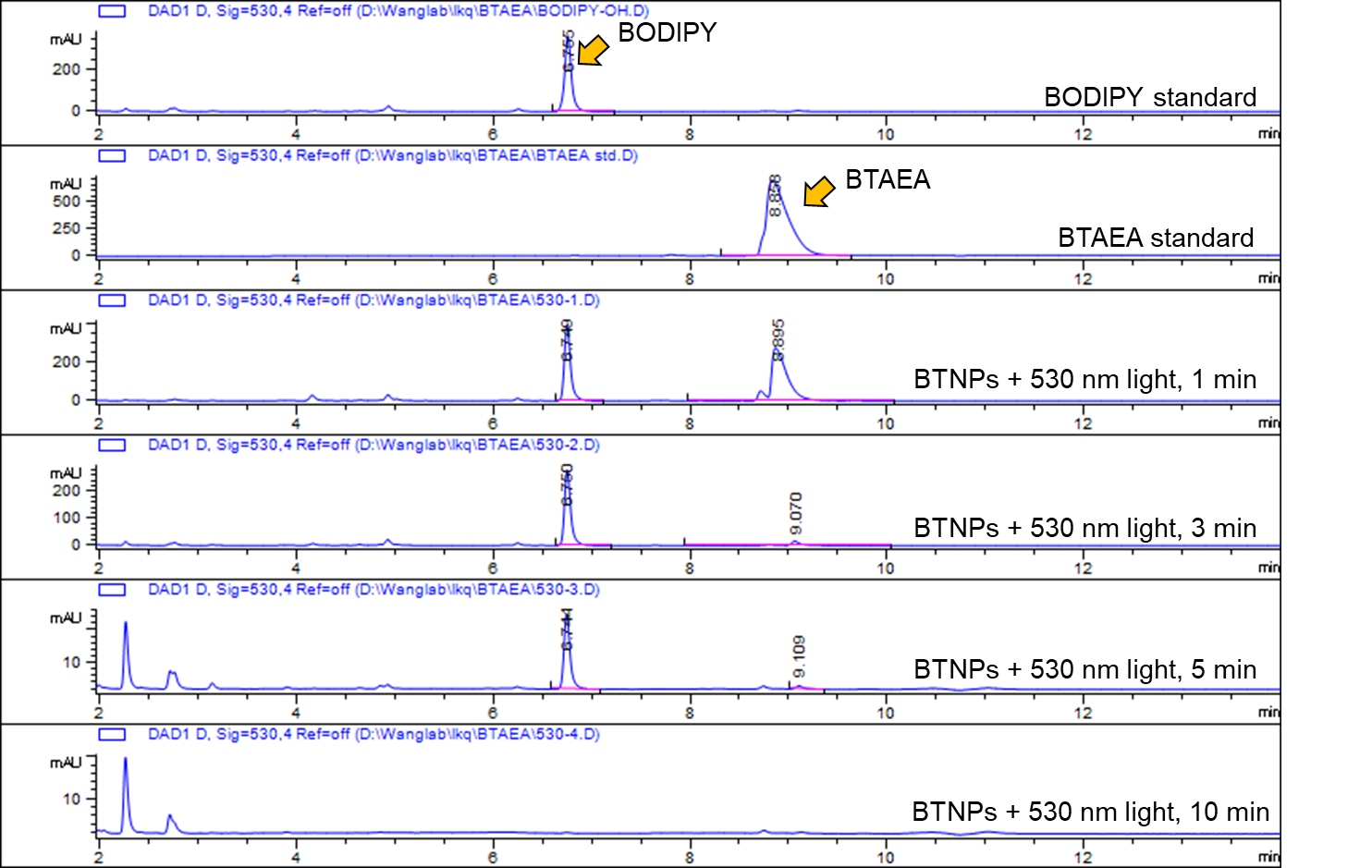


**Figure S14.** Photolysis of BTAEA in BTNPs with 530 nm light irradiation (50 mW/cm^2^).


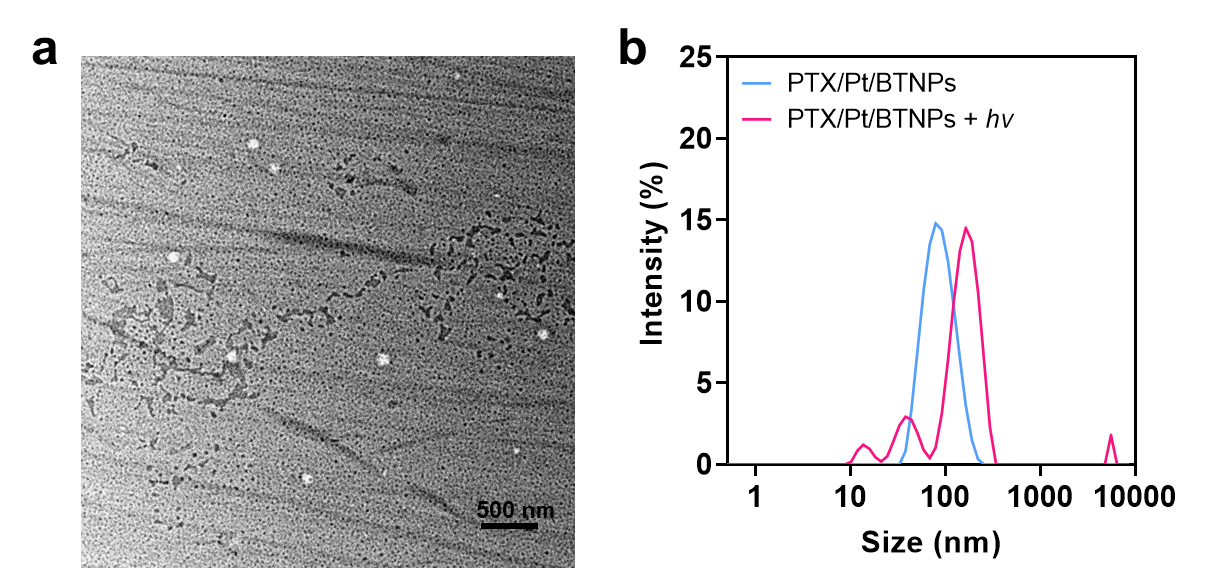


**Figure S15.** TEM image and DLS data of PTX/Pt/BTNPs in aqueous solutions after red light irradiation (635 nm, 50 mW/cm^2^, 10 min).


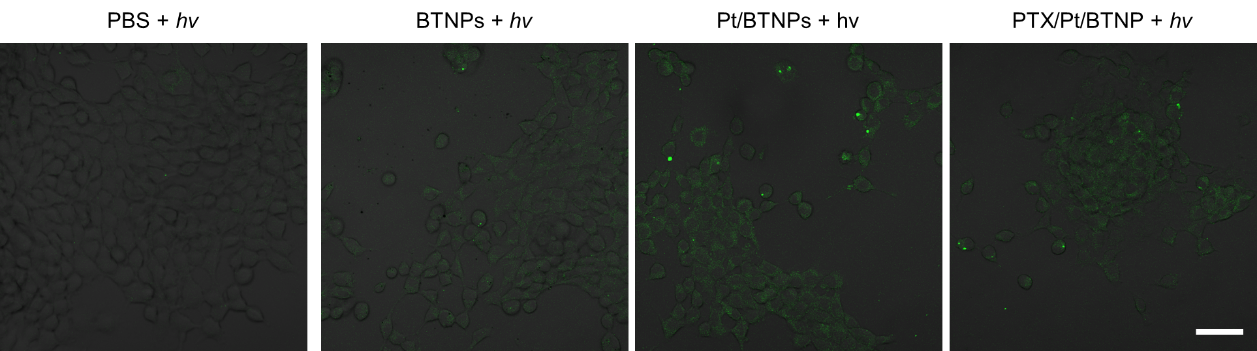


**Figure S16.** CLSM images of intracellular ROS generation in 4T1 cells treated with PBS, BTNPs, Pt/BTNPs and PTX/Pt/BTNPs, separately. The concentration was set as 5 μg/mL on BTAEA basis. DCFH-DA (10 μM) was used as an indicator for intracellular ROS. Light irradiation condition: 635 nm, 50 mW/cm^2^,10 min. Scale bar: 50 μm.


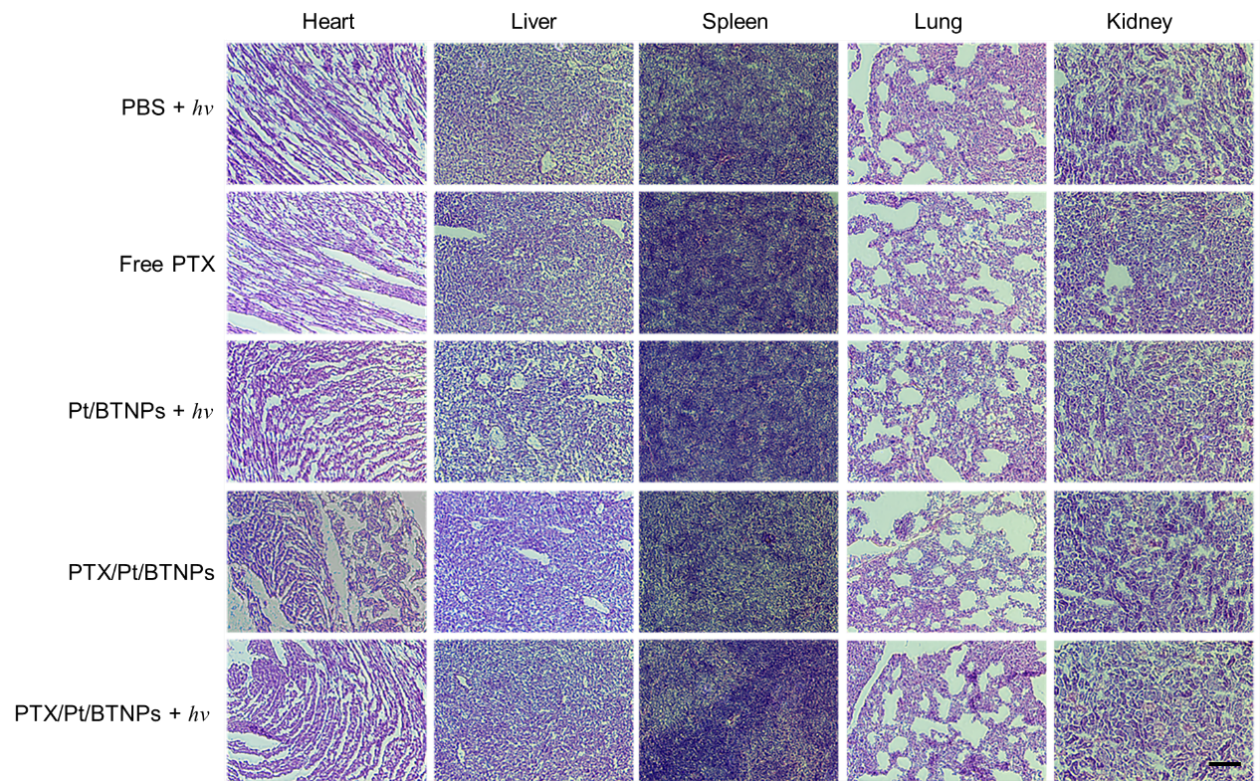


**Figure S17.** Representative photomicrographs of hematoxylin & eosin-stained sections of heart, lung, spleen, liver and kidney from different treatment groups. Scale bar: 200 µm.

**Reference**

1. W. Lv, Y. Li, F. Li, X. Lan, Y. Zhang, L. Du, Q. Zhao, D. L. Phillips, W. Wang. Upconversion-like photolysis of bodipy-based prodrugs via a one-photon process. *J Am Chem Soc*. **141**(44), 17482-17486 (2019).
